# Estimand Alignment and Implementation Sensitivity in Causal Mediation Analysis of Environmental Mixtures: Simulation, Plasmode, and Applied Study

**DOI:** 10.3390/stats9060121

**Published:** 2026-10-30

**Authors:** Augustina Odediran, Emmanuel Obeng-Gyasi

**Affiliations:** 1 Department of Built Environment, North Carolina A&T State University, Greensboro, NC 27411, USA; 2 Environmental Health and Disease Laboratory, North Carolina A&T State University, Greensboro, NC 27411, USA

**Keywords:** causal mediation analysis, environmental mixtures, estimand alignment, Bayesian kernel machine regression, Bayesian additive regression trees, plasmode simulation, PFAS, metals, dietary inflammatory index, liver, 62D20, 62F15, 62G08, 62P10, 62P12

## Abstract

Causal mediation analysis of environmental mixtures is challenging because exposures are correlated and mediation effects depend on joint exposure intervention. We compared Bayesian kernel machine regression causal mediation analysis (BKMR-CMA) with Bayesian additive regression trees causal mediation analysis (BART-CMA) for a simultaneous shift of mixture components from the 25th to the 75th percentile in seventeen synthetic scenarios and two plasmode simulations based on National Health and Nutrition Examination Survey (NHANES) data. We evaluated DeepMed separately because its binary treatment formulation could not target the same continuous mixture intervention. With an exposure–mediator interaction, a binary outcome, or additional effect-carrying components, BKMR-CMA had smaller indirect-effect bias and better interval coverage than BART-CMA; without interaction, BART-CMA was approximately unbiased. BART-CMA’s bias did not grow with the number of components when the added components were inert but increased markedly when they carried additional effects. In the NHANES-based plasmode, the ordering reversed: BART-CMA was more accurate, BKMR-CMA’s mean indirect effect had the wrong sign, and neither method reliably detected the imposed indirect effect. DeepMed performed poorly against both the continuous-mixture truth and the truth for its own binary contrast, so its error reflected estimator performance, not estimand mismatch alone. Among 1498 adults aged 20 years or older, BART-CMA estimated near-zero indirect effects of a PFAS–metal mixture on six liver outcomes through the Dietary Inflammatory Index. No estimator was uniformly superior: performance depended on estimand alignment, interaction structure, the number of effect-carrying components, the outcome scale, cross-world support, and implementation.

## 1. Introduction

Environmental epidemiology has shifted from studying individual toxicants to evaluating chemical mixtures, reflecting that real-world environmental exposures rarely occur in isolation [1]. This shift also poses a fundamental challenge for causal mediation analysis, which must accommodate correlated, potentially interacting exposures and a clearly defined multivariate intervention. Humans are simultaneously exposed to multiple environmental contaminants, including per- and polyfluoroalkyl substances (PFASs) and heavy metals such as lead, cadmium, and mercury. These chemicals are widespread, can bioaccumulate, and have been associated with adverse metabolic outcomes, including hepatotoxicity [2].

Epidemiologic studies have reported positive associations between serum concentrations of PFASs, particularly perfluorooctane sulfonate (PFOS) and perfluorooctanoic acid (PFOA), and biomarkers of hepatocellular injury, including alanine aminotransferase (ALT) and gamma-glutamyl transferase (GGT). In multivariable-adjusted analyses, PFOS, PFOA, perfluorononanoic acid (PFNA), and perfluorohexane sulfonic acid (PFHxS) have also been associated with altered alkaline phosphatase (ALP), aspartate aminotransferase (AST), and bilirubin concentrations, and obesity, high-fat dietary patterns, and systemic inflammation may modify these associations [3–6]. Exposure to heavy metals may likewise promote oxidative stress and subclinical liver injury. Cadmium, lead, and mercury have been linked to hepatic oxidative stress and inflammation, which may contribute to hepatocellular injury and, in some cases, hepatic steatosis and fibrosis. Proposed mechanisms include reactive oxygen species generation, mitochondrial dysfunction, and disruption of autophagy [7,8]. However, the biological pathways through which exposure mixtures may influence liver disease and hepatic biomarkers remain poorly understood.

These exposures may jointly influence health outcomes through complex, nonlinear interactions and shared biological pathways, including systemic inflammation [9–11]. One biologically plausible mechanism linking environmental toxicant burden to liver pathogenesis is systemic inflammation, which dietary patterns may substantially influence. The Dietary Inflammatory Index (DII) is a validated tool that quantifies the inflammatory potential of an individual’s overall dietary intake [12]. Because environmental toxicants can disrupt metabolic homeostasis and diet acts as a critical interface in the body’s systemic inflammatory response, DII is a plausible mediator linking exposure to liver outcomes such as ALT, AST, GGT, ALP, liver fat, and fibrosis indices [3,4,13]. Understanding what proportion of a chemical mixture’s total effect on liver disease operates through diet-induced inflammation (the natural indirect effect) versus direct hepatotoxic pathways (the natural direct effect) is important for developing targeted public health and nutritional interventions.

Traditional parametric mediation models assume linear relationships and no exposure–exposure interactions, assumptions that are ill-suited to high-dimensional, correlated mixtures with complex dose–response surfaces. Recent methodological work has therefore introduced flexible, nonparametric and semiparametric approaches that accommodate these complexities while preserving causal interpretability [14–16]. Across application domains such as environmental mixtures, genomic intervention, and high-dimensional omics, these methods aim to decompose a total effect into direct and indirect components via one or more mediators, while imposing weaker parametric constraints and enabling robust inference in high-dimensional settings [17–19].

We evaluated three approaches to mediation analysis with multiple environmental toxicants measured simultaneously. These methods differ in their assumptions, modeling strategies, and estimands.

Bayesian kernel machine regression causal mediation analysis (BKMR-CMA) is a flexible semiparametric framework for estimating causal mediation effects, including natural direct, natural indirect, and controlled direct effects, in complex environmental mixture settings [14]. By combining parametric covariate adjustment with Gaussian process kernels, BKMR-CMA models nonlinearities and high-dimensional interactions among mixture components nonparametrically in both the mediator and outcome models. It uses Markov chain Monte Carlo (MCMC) posterior-predictive simulation from joint fits to perform counterfactual inference, providing an approach for evaluating joint and individual mixture-mediated pathways [20].

Bayesian additive regression trees (BART) is a nonparametric Bayesian machine learning method that models the target function as an ensemble sum of regression trees, offering flexibility in estimating complex exposure–response surfaces [21]. Recently extended to environmental mixtures and causal mediation frameworks, BART has become a widely used tool in causal inference. Its regularization priors control tree depth and leaf-node parameters, allowing it to capture high-dimensional interactions and nonlinearities among exposures, mediators, and outcomes without parametric specification [22,23].

DeepMed uses deep neural networks to estimate nuisance functions within a semiparametric framework. Built on Neyman-orthogonal efficient influence functions and cross-fitting, it achieves robust asymptotic properties without network sparsity constraints [24]. However, bridging the gap between its theoretical efficiency and its empirical performance remains challenging because of sample size requirements, hyperparameter sensitivity, and susceptibility to positivity violations.

Despite the theoretical advantages of these estimators, a gap remains in understanding their comparative performance under the data structures characteristic of environmental epidemiology, namely highly correlated multi-component exposures, continuous mediators, and skewed clinical biomarkers. The objectives of this study were therefore twofold:

- To conduct a simulation study comparing the bias, root mean squared error (RMSE), interval coverage, and computation time of BKMR-CMA, BART-CMA, and DeepMed under data-generating processes characterized by high multicollinearity, nonlinearity, and varying interaction depth;
- To apply the method identified as best suited to the applied data structure to estimate the mediating role of DII in the association between a chemical mixture (PFOA, PFOS, lead, mercury and cadmium) and liver biomarkers (liver fibrosis, liver fat, ALT, AST, ALP, and GGT), thereby examining the inflammatory pathways proposed to drive environmentally induced hepatotoxicity.

## 2. Materials and Methods

### 2.1. Overview

We conducted a Monte Carlo simulation study to compare three flexible approaches for estimating natural direct and indirect effects of a multi-pollutant exposure mixture: BKMR-CMA, BART-CMA, and DeepMed. The study was designed and reported in accordance with the ADEMP framework [25]. The aim was twofold: to determine which estimator most accurately recovers the mixture-level natural indirect effect, as assessed by bias and nominal interval coverage, and to identify the data-generating conditions under which each estimator performs poorly or fails. Each method targets a different estimand, and comparison requires that all methods target the same causal estimand under an identical exposure contrast. We refer to this requirement as **estimand alignment** and describe it in Section 2.4.

### 2.2. Notation

Throughout, bold symbols denote vectors and matrices, italic symbols denote scalars, and all vectors are column vectors. Let $Z={\left(Z_{1},\ldots ,Z_{L}\right)}^{⊤}$ denote a mixture of L correlated exposures, M a continuous mediator, Y the outcome, and $C={\left(C_{1},C_{2},C_{3}\right)}^{⊤}$ a vector of measured confounders (Figure 1), and let $1_{k}$ denote a k-vector of ones. Let Y(z,m) be the potential outcome under exposure z and mediator value m, M(z) the potential mediator under exposure z, and $Y(z,M(z^{\prime }))$ the potential outcome under exposure z when the mediator takes the value it would have taken under $z^{\prime }$.

### 2.3. Data-Generating Mechanism

#### Confounders and exposures.

Three confounders were drawn as $C\sim N_{3}\left(0,\Sigma_{C}\right)$, with unit variances and exchangeable pairwise correlation 0.2. Exposures were then generated conditionally on the confounders,

(1) $$Z=\theta_{CZ}\left(1_{3}^{⊤}C\right)\;1_{L}+\varepsilon_{Z},\;\varepsilon_{Z}\sim N_{L}\left(0,\Sigma_{Z}\right),$$

so that each exposure depends on the sum of the confounders, $Z_{j}=\theta_{CZ}\left(C_{1}+C_{2}+C_{3}\right)+\varepsilon_{Zj}$, with $\theta_{CZ}=0.1$. The covariance matrix $\Sigma_{Z}$ has unit variances and exchangeable pairwise correlation $\rho_{Z}=0.3$ (0.7 in Scenario 17 and a block structure in Scenario 18; Section 2.5). Because C enters the exposure, mediator, and outcome models, it is a genuine confounder of all three relationships rather than a precision covariate.

#### Mediator.

The mediator was generated as

(2) $$M=\alpha_{0}+h_{M}(Z)+\alpha_{C}^{⊤}C+\varepsilon_{M},\;\varepsilon_{M}\sim N(0,\;1),$$

with $\alpha_{0}=0.5$ and $\alpha_{C}={\left(0.3,-0.2,\;0.1\right)}^{⊤}$. Under the linear setting, $h_{M}(Z)=\alpha_{Z}^{⊤}Z$. Under the nonlinear setting,

(3) $$h_{M}(Z)=\alpha_{Z1}Z_{1}+0.15\;\kappa Z_{1}^{2}+\alpha_{Z2}\;sign\left(Z_{2}\right)\sqrt{\left|Z_{2}\right|}+\sum_{j=3}^{L}\alpha_{Zj}Z_{j},$$

where $\alpha_{Zj}$ is the *j*th element of $\alpha_{Z}$ and κ scales the strength of the nonlinear terms, with κ=1 except in Scenario 19, where κ=2 (Section 2.5). Nonlinearity is thus confined to the two most strongly weighted exposures, while the remainder enter linearly.

#### Outcome.

The outcome linear predictor was

(4) $$\eta =\beta_{0}+h_{Y}(Z)+\beta_{M}M+\delta \left(\beta_{ZM}^{⊤}Z\right)M+\beta_{C}^{⊤}C,$$

where δ=1 in scenarios with exposure-mediator interaction and δ=0 otherwise, $\beta_{0}=1.0$, $\beta_{M}=0.5$, and $\beta_{C}={\left(0.2,-0.1,\;0.15\right)}^{⊤}$. Under the linear setting, $h_{Y}(Z)=\beta_{Z}^{⊤}Z$; under the nonlinear setting,

(5) $$h_{Y}(Z)=\beta_{Z1}Z_{1}+0.1\;\kappa Z_{1}^{2}+\beta_{Z2}\;sign\left(Z_{2}\right)\sqrt{\left|Z_{2}\right|}+0.15\;\kappa Z_{1}Z_{2}+\sum_{j=3}^{L}\beta_{Zj}Z_{j},$$

which adds a two-way exposure–exposure product absent from the mediator model. Continuous outcomes were generated as

(6) $$Y=\eta +\varepsilon_{Y},\;\varepsilon_{Y}\sim N(0,1),$$

and binary outcomes as

(7) Pr(Y=1∣Z,M,C)=Φ(η/1.7),

a scaled probit that approximates a logistic link while remaining conjugate to the probit machinery used by both Bayesian estimators.

#### Coefficients across mixture dimensions.

We defined coefficient vectors once at length 10 and truncated them to the first L elements. Hence, scenarios differing in L share their leading coefficients and differ only by the addition or removal of trailing exposures.

The base vectors were $\alpha_{Z}={\left(0.6,0.4,0.3,0.2,0.1,0.5,0.35,0.25,0.15,0.05\right)}^{⊤}$, $\beta_{Z}={\left(0.4,0.3,0.2,\;0.1,0.05,0.35,0.25,0.15,0.08,0.03\right)}^{⊤}$, and $\beta_{\mathrm{ZM}}={\left(0.2,0.15,0.1,0.05,0,0.18,0.12,0.08,0.03,0\right)}^{⊤}$.

The L = 3, L = 5, and L = 10 scenarios are therefore nested rather than independently parameterized. Scenarios 15–16 relax this nesting by increasing L while holding the signal fixed (Section 2.5.2).

### 2.4. Estimands and Estimand Alignment

Because the exposure is a vector, a mediation effect is defined only relative to a stated shift in the whole mixture, following standard practice in the mixtures literature [14,26]. We fixed a single contrast across all scenarios and methods: a simultaneous shift of each mixture component from its 25th to its 75th percentile. Writing the reference and comparative exposure vectors as

(8) $$z_{t}={\left(q_{p_{t}}\left(Z_{1}\right),\ldots ,q_{p_{t}}\left(Z_{L}\right)\right)}^{⊤},\;t\in \{0,\;1\},\;p_{0}=0.25,\;p_{1}=0.75,$$

where $q_{p}\left(Z_{j}\right)$ is the p th quantile of the marginal distribution of $Z_{j}$, the three estimands are

(9) $$TE=E\left\{Y\left(z_{1},M\left(z_{1}\right)\right)\right\}-E\left\{Y\left(z_{0},M\left(z_{0}\right)\right)\right\},$$

(10) $$NDE=E\left\{Y\left(z_{1},M\left(z_{0}\right)\right)\right\}-E\left\{Y\left(z_{0},M\left(z_{0}\right)\right)\right\},$$

(11) $$NIE=E\left\{Y\left(z_{1},M\left(z_{1}\right)\right)\right\}-E\left\{Y\left(z_{1},M\left(z_{0}\right)\right)\right\},$$

so that TE = NDE + NIE. Identification follows the standard assumptions for natural effects: no unmeasured exposure–outcome, mediator–outcome, or exposure–mediator confounding given C; no mediator–outcome confounder affected by exposure; positivity; and cross-world independence, $Y(z,m)⊥M(z^{\prime })|C$ for all z, $z^{\prime }$ and m [26–28]. Figure 1 shows the causal structure assumed throughout, including the downstream cardiometabolic covariates adjusted for only in the sensitivity analysis of Section 2.10.7. Positivity in mediation additionally requires adequate support in the cross-world region, a condition that is testable in observed data [29] and that we report as a diagnostic in Section 2.5.1 [30]. By construction, the simulated data satisfy all of these conditions, so any departure of an estimate from truth is attributable to the estimator or to finite-sample support rather than to identification failure.

The three methods do not natively target this contrast. BKMR-CMA and BART-CMA can be configured to target the Q_25_ → Q_75_ mixture shift directly. DeepMed requires a binary treatment and cannot represent a continuous multivariate shift. This restriction applies only to the treatment; the mediator and outcome may be continuous or binary, so the limitation is one of exposure representation rather than of outcome type.

To apply DeepMed in this setting, we binarized the unweighted mixture index $S=\sum_{j=1}^{L}Z_{j}$ in two ways. First, we used a median split, with low and high exposure levels defined as the mean exposure vectors within each half of the sample; this is the native DeepMed configuration and retains all observations. Second, we constructed a Q_75_-versus-Q_25_ contrast by retaining only individuals above the 75th or below the 25th percentile of the index and discarding the middle half, yielding *n* = 150 when *n* = 300. In both cases, the exposure levels are defined from the realized index distribution rather than from a fixed componentwise shift, so the actual contrast varies across replicates and was recorded in each scenario. Averaged over replicates in scenario 1, the median split produced a mean componentwise contrast of 1.11 and the quartile binarization 1.77, against 1.38 for the target Q_25_ → Q_75_ shift. The two binarizations therefore depart from the target in opposite directions: the median split is too narrow and the quartile contrast too wide, so neither is more closely aligned with the target than the other.

DeepMed estimates were evaluated primarily against a common reference, defined by the componentwise Q_25_ to Q_75_ mixture shift, ensuring comparability with other estimators. To distinguish the two sources of error inherent in this comparison, DeepMed was also evaluated against the reference corresponding to the contrast targeted by its binarization: the shift from the mean exposure vector of the lower group to that of the upper group, defined as below versus above the median of the mixture index, or below the 25th versus above the 75th percentile. Both references were computed from the same 10^6^ simulated observations. The difference between these two references quantifies the estimand mismatch, while the difference between the DeepMed estimate and its corresponding reference quantifies estimation error. Conceptually, the index-based contrasts are not equivalent to the target mixture estimand: the index splits individuals by their realized sum of exposures, whereas the target contrast imposes an equal percentile shift on each component, and individuals with identical index values may differ substantially in their exposure profiles. In addition, the DeepMed covariate set includes the full exposure vector Z, while the binary treatment indicator is a deterministic function of Z; conditioning on Z therefore removes most variation in the treatment indicator, directly affecting the positivity condition.

For these reasons we pre-specified that DeepMed results in continuous-outcome scenarios would not be pooled with BKMR-CMA and BART-CMA in the primary performance comparison, and would instead be reported separately with the estimand mismatch explicitly labeled (Section 2.5.1).

#### Scale Commensurability

Estimand alignment concerns not only the exposure contrast but also the scale on which effects are expressed. For binary outcomes, both Bayesian estimators use probit models whose regression surfaces are defined on a latent scale. In contrast, the estimands defined above are risk differences on the outcome-probability scale. Counterfactual contrasts formed before application of the probit link are therefore not commensurable with the Monte Carlo truth. In our binary-outcome scenarios, latent-scale contrasts inflated the NDE, NIE, and TE by factors of approximately 3.4 to 3.8.

We therefore applied the probit link to each posterior-predictive draw before averaging the counterfactual outcomes and forming effect contrasts. This ordering matters because the probit link is nonlinear: transforming an averaged latent-scale prediction is not generally equivalent to averaging predictions after transformation to the probability scale. Latent-scale estimates may nevertheless appear plausible, having the expected direction and relative ordering of effects, which makes the scale mismatch difficult to identify from model output alone. We discuss the distinction between latent-scale and risk-difference estimands for binary mediation in detail elsewhere [31].

### 2.5. Simulation Design

The simulation design comprised 17 scenarios: an eight-scenario fully crossed core and four targeted extension arms (Table 1). The core scenarios (1–8) crossed mediator and outcome functional form (linear versus nonlinear), exposure–mediator interaction (absent versus present), and sample size (*n* = 300, *n* = 1000), while holding the outcome continuous and the mixture dimension at L = 5. We selected these factors because each represents a plausible challenge for the estimators under comparison. Kernel and tree ensembles may differ in their ability to approximate smooth and non-smooth response surfaces; exposure–mediator interaction makes the cross-world component of the NIE dependent on extrapolation; and sample size determines whether flexible models have sufficient information to distinguish direct and mediated pathways.

The outcome-type extension (scenarios 9–10) repeated the most demanding core condition, nonlinear functional forms with an exposure–mediator interaction, using a binary outcome at *n* = 300 and *n* = 1000. The mixture-size extension (scenarios 11–12) repeated this same condition with a continuous outcome at *n* = 300, varying the number of mixture components between L = 3 and L = 10. A fixed-signal arm (scenarios 15–16) repeated this condition at L = 5 and L = 10 with the mediator, outcome, and interaction coefficients held at the L = 3 specification (Section 2.5.2). An exposure-correlation and nonlinearity arm (scenarios 17–19) changed one further feature of this condition at a time (Section 2.5.3). All four extension arms were therefore anchored to the nonlinear, interaction-present condition, allowing observed performance differences to be attributed to outcome type, mixture size, signal, exposure correlation, or strength of nonlinearity within that challenging setting.

The design was not fully crossed across all five factors because the resulting grid was computationally infeasible: BKMR-CMA runtime exceeded 24 h per scenario at *n* = 1000. Consequently, we evaluated outcome type and mixture dimension only within the nonlinear, interaction-present condition. We cannot interpret them as marginal main effects across other functional-form or interaction settings.

#### 2.5.1. Plasmode Arm (Scenarios 13–14)

The synthetic scenarios share a feature that limits their direct relevance to the applied analysis. Across Scenarios 1–12 and 17–19, the induced correlation between the mixture index and the mediator ranged from 0.66 to 0.85 (0.57–0.61 in Scenarios 15–16, whose mixture index includes inert exposures), whereas the corresponding correlation in the NHANES application described in Section 2.10 was −0.06. Likewise, occupancy of the cross-world region, the proportion of observations jointly in the upper exposure quartile and the lower mediator quartile, a region relevant to estimation of the NIE, ranged from 0.04% to 0.78% across Scenarios 1–12 and 17–19 (0.98% and 1.42% in Scenarios 15–16) compared with 6.8% in the applied data. The synthetic design therefore evaluates estimator performance under an exposure–mediator support geometry that differs materially from that observed in the application, and its findings should be interpreted as evidence about performance under the simulated conditions. Because the target intervention shifts each component separately, a scalar mixture index summarizes the exposure vector along a single direction and can misstate the support available for the counterfactual points that the NIE requires. We therefore also measured support directly in the joint (L + 1)-dimensional exposure–mediator space. The NIE requires outcome predictions at ($z_{1},\;m$), where $z_{1}$ is the vector of componentwise 75th percentiles and m is a mediator value that would occur under the vector of 25th percentiles, $z_{0}$. For each observed confounder profile $c_{i}$, we drew ${\;m}_{0i}$ from the mediator distribution under $z_{0}$ given $c_{i}$, using the true mediator model in the synthetic and plasmode arms and, in the NHANES data, a linear model for DII given the exposures and confounders fitted in each imputed dataset, and formed the cross-world point ($z_{1},{\;m}_{0i}$). After whitening the observed (Z, M) by its sample covariance, so that distances are Mahalanobis distances that account for the correlation among exposures and between exposures and the mediator, we computed the distance from each cross-world point to its fifth-nearest observed point and classified the point as outside the observed support if this distance exceeded the 95th percentile of the same leave-one-out distance among the observed points. By construction, about 5% of points drawn from the observed distribution are classified as outside (5.3% in a calibration check with independent draws from the Scenario 7 model). As a comparator, we applied the same rule to the factual points $\left(z_{1},{\;m}_{1i}\right)$ and $\left(z_{0},{\;m}_{0i}\right)$.

Factual points were almost never classified as outside the observed support (at most 0.3% in any setting). By contrast, 8.9% to 11.3% of cross-world points lay outside the observed support in Scenarios 1–11 and 17–19, and 32.0% in Scenario 12, against 0.05% to 0.06% in the plasmode cells and 0.2% in the NHANES data (Figure 2; Table S5). The scalar and multivariate measures ranked the 20 settings consistently (Spearman ρ = −0.66), and every synthetic scenario had less support than the NHANES-based data, a result that held with the first or tenth nearest neighbor and with a 99th-percentile threshold. The multivariate measure describes overlap, not estimator error. In Scenarios 15 and 16, where the added exposures were inert, it classified fewer cross-world points as outside the observed support (5.2% and 0.6%), yet the relative NIE bias of BART-CMA (−29% and −33%) was similar to that in Scenario 11 (−32%; 11.3% outside). Exposures that carry no signal enlarge nearest-neighbour distances among the observed points as well as for the cross-world points, so a distance computed over all components can understate the sparsity that matters for the components that drive the mediator and outcome. We therefore report the two measures together.

To address this limitation, we included two plasmode scenarios based on the empirical exposure and confounder structure of the applied dataset. Plasmode simulation resamples observed exposures and covariates without modification, preserving their observed joint dependence structure, while simulating the mediator and outcome from investigator-specified structural equations with known causal effects [32]. This approach is increasingly used to evaluate causal-inference methods when realistic covariate dependence and overlap are central to the scientific setting [33]. Each replicate drew *n* = 1000 rows without replacement from the analytic dataset of *n* = 1498, using the first of the 20 multiply imputed datasets as a fixed, documented base. We fitted the mediator and outcome equations to the observed data to obtain coefficient signs and the relative structure. We imposed an exposure–mediator interaction at half the magnitude of the corresponding direct exposure coefficients, and we centered the mediator in the outcome equation. Centering is required, not cosmetic: with an uncentred mediator, the interaction term adds a quantity proportional to the mediator’s mean to the direct effect, which, for a mediator with a mean of 1.60, inflates the NDE by orders of magnitude.

Scenario 13 retains the fitted coefficients, yielding a true NIE of −0.0001 outcome standard deviations (−0.00005 on the outcome scale), an effectively null mediated pathway; it tests whether the estimators return a null under realistic support geometry. Scenario 14 amplifies the exposure–mediator path threefold and solves for the mediator–outcome coefficient such that the true NIE equals −0.10 outcome standard deviations, testing whether a non-null indirect effect is recovered. Because the standard deviation of the observed outcome is 0.394, the imposed effect corresponds to a true NIE of −0.0394 on the outcome scale; all performance measures for the plasmode cells are reported on that scale rather than in standard deviation units. Together, the two cells distinguish correct null recovery from insensitivity, which neither can do alone.

The mediated pathway in these data is weak: exposures explain 2.4% to 3.5% of mediator variance across all twenty imputations, and the Q_25_ → Q_75_ contrast shifts the mediator by less than 0.07 standard deviations. A non-null indirect effect cannot be studied without departing from the fitted magnitudes. Imposing an effect size is standard within the plasmode framework, where the realism claim attaches to the covariate structure rather than to the coefficients [32]. We state the departure explicitly rather than presenting the imposed effects as estimates. In the plasmode cells, the mixture–mediator correlation is −0.05 and −0.06 and cross-world occupancy is 6.8% and 7.2%, compared with −0.06 and 6.8% in the applied data.

We ran the plasmode cells as a separate workflow and merged them at the analysis stage. They are not part of the factorial design and are not marginalized with the seventeen synthetic scenarios. Because the outcome model is linear in the mediator and the intervention contrast is fixed, the true NIE in these cells is analytically invariant to which rows are resampled; across 200 replicates, its Monte Carlo standard deviation was below 1 × 10^−16^ . The TE and NDE vary through the finite-population confounder distribution. We therefore computed truth per replicate for the TE and NDE, and it is constant for the NIE. This is a property of the design rather than a limitation: bias in the NIE cannot be confounded with variation in the target.

#### 2.5.2. Fixed Signal Mixture Size Arm (Scenarios 15–16)

We truncated the coefficient vectors as described in Section 2.3, causing Scenarios 11 and 12 to differ in both mixture size and the number of exposures with nonzero mediator, outcome, and interaction coefficients. Consequently, the true NIE increases from 1.55 at L = 3 to 4.63 at L = 10, so we cannot attribute performance differences to mixture size alone. To disentangle these factors, we added Scenarios 15 and 16, which reproduce the conditions of Scenarios 11–12—nonlinear functional forms, exposure–mediator interaction, a continuous outcome, and *n* = 300—at L = 5 and L = 10, respectively. In both scenarios, we retained the original values of the first three elements of $\alpha_{Z}$, $\beta_{Z}$, and $\beta_{ZM}$ and set all remaining coefficients to zero. We generated additional exposures using the same mechanism as the active exposures, preserving their marginal distribution, dependence on confounders, and pairwise correlation of approximately 0.33 with every other exposure. These additional exposures therefore share the distributional characteristics of the active exposures but do not enter the mediator or outcome models. This construction preserves the true effects of Scenario 11, and our numerical calculations yielded NIE values that agreed within 0.003. Comparisons among Scenarios 11, 15, and 16 isolate the effect of increasing mixture size while holding signal constant, whereas the comparison between Scenarios 16 and 12 isolates the effect of increasing signal at a fixed mixture size (L = 10). This design evaluates sparse mixtures with additional inert exposures; we did not examine dense mixtures in which many components contribute small effects. We used the same estimators, settings, and replicate seeding as in the other synthetic scenarios.

#### 2.5.3. Exposure Correlation and Nonlinearity Arm (Scenarios 17–19)

Three further scenarios each change one feature of Scenario 7 (nonlinear mediator and outcome models with exposure–mediator interaction, *n* = 300, five exposures, continuous outcome). In Scenario 17, the exchangeable correlation among the exposures conditional on the confounders was raised from 0.3 to 0.7. In Scenario 18, the exposures had a block structure resembling the applied mixture, in which the two PFASs were more strongly correlated with each other than the metals were: the first two exposures were correlated at 0.7, the remaining three at 0.3, and exposures in different blocks at 0.1. In Scenario 19, the quadratic terms and the $Z_{1}Z_{2}$ interaction term of both the nonlinear mediator model and the nonlinear outcome model were doubled, with all other coefficients unchanged (Table 1). True effects were computed as for the other scenarios (Section 2.6), and each scenario was analyzed with 100 replications.

### 2.6. True Values

We computed true NDE, NIE, and TE by Monte Carlo integration under the known structural equations. We computed the contrast vectors $z_{0}$ and $z_{1}$ of Equation (8) from 10^6^ simulated observations. We then obtained effects by integrating over 10^5^ draws of the confounder vector and the mediator’s stochastic variation, holding exposure fixed at $z_{0}$ and $z_{1}$ as the counterfactual requires. We evaluated outcomes at their conditional mean for continuous scenarios or conditional probability for binary scenarios, so no outcome-level noise enters the truth. The decomposition TE = NDE + NIE holds exactly by construction.

Because the estimands are population quantities, the truth depends only on linearity, interaction, outcome type, and mixture dimension; scenarios differing solely in sample size share a common truth. We computed truth values once per distinct parameter configuration under a fixed seed and cached them. The truth marginalizes over the confounder distribution. For continuous outcomes, this is equivalent to evaluating at the confounder mean, because confounders enter the mediator and outcome models linearly and additively and their contribution cancels in every effect contrast. For binary outcomes, the link is nonlinear, and the two are not equivalent, which is one reason the binary scenarios are reported separately (Section 2.8).

### 2.7. Methods and Implementation

We analyzed each of the 100 replicate datasets per scenario with all applicable methods, so comparisons are paired. We generated datasets from a replicate-specific seed determined by scenario and replicate index, ensuring every method saw identical data.

#### 2.7.1. BKMR-CMA

BKMR-CMA embeds Bayesian kernel machine regression [34,35] within the mediation formula, simulating counterfactuals from the posterior predictive distributions of the mediator and outcome [14]. We fitted three kmbayes models per replicate: a mediator model, an outcome model including the mediator in the kernel, and an exposure-only model for the total effect, each with 3000 MCMC iterations, the first 1500 discarded as burn-in. We computed the natural direct and total effects directly, and obtained the natural indirect effect by subtraction: NIE=TE-NDE.

##### Kernel approximation.

A Gaussian predictive process approximated all kernels [36], with 100 knots selected by space-filling design (fields::cover.design) [37], an option provided by the bkmr implementation for large datasets [34]. Knots were constructed in each kernel’s own space: the exposure space for the mediator and total-effect models, and the joint exposure–mediator space for the outcome model. The approximation was applied uniformly across all scenarios to avoid confounding by outcome type, sample size, or mixture dimension. We validated it against exact-kernel fits at *n* = 300, where it altered the estimated NIE by 0.006 outcome standard deviations; at *n* = 1000 with a binary outcome, the exact kernel was numerically singular in 100 of 100 replicates, so the approximation is required rather than optional at that sample size.

##### Variable selection.

Componentwise variable selection [35] was enabled for continuous outcomes. For binary outcomes, it caused numerical failure in the probit model under the final implementation (three of five Scenario 9 fits failed in a pilot check) and was therefore disabled, so the binary arm estimates BKMR-CMA rather than BKMR-CMA-VS; both are named variants [14]. To separate outcome type from this change in specification, we reran scenarios 1, 7, and 8 with variable selection disabled, so that scenarios 7–8 could be compared with their binary counterparts (scenarios 9–10) under an identical BKMR-CMA specification (Section 3.1.6).

##### Counterfactual construction and scale.

For each of 100 retained posterior samples, we drew 100 mediator values from the mediator model’s posterior predictive distribution and averaged the resulting counterfactual outcomes. For binary outcomes, the probit link was applied to each posterior draw before forming contrasts, so that the reported effects are risk differences commensurable with the truth (Section 2.4). Counterfactuals were evaluated at the mean confounder vector, following the method as specified by [14]. Under a nonlinear link, this differs from marginalizing over the confounder distribution as the truth does; we quantified the discrepancy directly under the known structural equations at 1.4% of the true NIE and retained the method’s native specification rather than modifying it.

##### Software correction.

The released causalbkmr package (version 0.1.0, commit 4a33c0b) contains an error in the internal function that computes the cross-world quantity $E\left\{Y\left(z_{1},M\left(z_{0}\right)\right)\right\}$. In step 2a of the published algorithm [14], for posterior sample j and . In step 2a of the published algorithm [14], for posterior sample j and mediator draw k, the counterfactual mediator is drawn from the mediator model evaluated at the reference exposure $z_{0}$,

(12) $$M^{\left(j,k\right)}\left(z_{0}\right)=h_{M}^{\left(j\right)}\left(z_{0}\right)+{\bar{c}}^{⊤}\alpha_{C}^{\left(j\right)}+\sigma_{M}^{\left(j\right)}\epsilon^{\left(j,k\right)},\;\epsilon^{\left(j,k\right)}\sim N(0,\;1),$$

where $h_{M}^{\left(j\right)}$, $\alpha_{C}^{\left(j\right)}$, and $\sigma_{M}^{\left(j\right)}$ are the j th posterior draws of the mediator model’s exposure–response function, confounder coefficients, and residual standard deviation, and $\bar{c}$ is the mean confounder vector at which counterfactuals are evaluated. The implementation instead evaluates the mediator model at the comparative exposure $z_{1}$; the reference-level argument is accepted by the function but never used in its body. The counterfactual mediator is therefore drawn under $z_{1}$, and the cross-world quantity is computed as $E\left\{Y\left(z_{1},M\left(z_{1}\right)\right)\right\}$.

(13) $$TE^{\left(j\right)}={\overset{ˆ}{Y}}_{TE}^{\left(j\right)}\left(z_{1}\right)-{\overset{ˆ}{Y}}_{TE}^{\left(j\right)}\left(z_{0}\right),$$

(14) $$NDE^{\left(j\right)}={\overset{ˆ}{Y}}^{\left(j\right)}\left(z_{1},M\left(z_{0}\right)\right)-{\overset{ˆ}{Y}}_{TE}^{(j}\left(z_{0}\right),$$

(15) $$NIE^{\left(j\right)}=TE^{\left(j\right)}-NDE^{\left(j\right)}={\overset{ˆ}{Y}}_{TE}^{\left(j\right)}\left(z_{1}\right)-{\overset{ˆ}{Y}}^{\left(j\right)}\left(z_{1},M\left(z_{0}\right)\right).$$

where ${\overset{ˆ}{Y}}^{\left(j\right)}(\cdot )$ is the counterfactual outcome averaged over the mediator draws and ${TE}^{\left(j\right)}$ is the total effect. With the cross-world term computed as ${\overset{ˆ}{Y}}^{\left(j\right)}\left(z_{1},M\left(z_{1}\right)\right)$, the estimated NDE becomes an estimate of the total effect, and the estimated NIE is identically/approximately zero. This holds in every scenario irrespective of sample size, effect magnitude, or model specification. The total effect and the controlled direct effects are computed on separate code paths and are unaffected.

The defect is not detectable from output alone: a null indirect effect is a substantively plausible result in mediation analysis, and the affected quantities retain the expected sign and relative ordering. We identified it by comparing package output against an independently written implementation of the same algorithm on identical data. We corrected the single line locally; following the correction, the package reproduced the independent implementation to within 0.01. We report this to support reproduction of our results and to note that any analysis using this commit is affected in the same way.

#### 2.7.2. BART-CMA

BART-CMA applies Bayesian additive regression trees [38] to the mediator and outcome models and computes natural effects by Bayesian g-formula, following Linero and Zhang [39]. We fitted separate mediator and outcome ensembles with 200 trees, 2000 burn-in iterations, and 1000 posterior draws, retaining 200 draws via systematic thinning for the g-computation step. For each retained draw, we simulated mediator values from that draw’s fitted mediator surface using that draw’s residual standard deviation; we drew and averaged five residual realizations within the draw; and we averaged the resulting counterfactual outcomes over the replicate’s empirical confounder distribution. All three effects were computed directly from the same pair of fits. We mapped binary outcomes to the probability scale before averaging.

The pairing of the mediator draw with the outcome draw is essential and is not implied by the algorithm as ordinarily described. An implementation that draws mediator values independently of the posterior index, for instance by drawing a single mediator realization and applying it across all posterior samples, averages mediator parameter uncertainty out of the posterior rather than propagating it into the interval. As a result, intervals are too narrow by construction, while point estimates are unaffected. On identical data, correcting this widened the mean NIE interval by 55% to 98% across scenarios 1–12 (0.602 to 1.131 in scenario 1) and changed mean bias by less than one Monte Carlo standard error in every cell. Interval-based performance measures reported below are from the corrected implementation. Mediator and outcome ensembles were fitted with the dbarts package in R 4.5.0; BKMR-CMA used the bkmr package together with the causalbkmr extension (github.com/zc2326/causalbkmr, commit 4a33c0b), and DeepMed used the DeepMed package (github.com/siqixu/DeepMed). The applied analysis scripts in the repository (01_impute_adults.R, run_applied_adults.sh, BART_CMA_Mediation.Rmd and 06_collect_applied.R) reproduce the imputation, the mediator and outcome fits, and the weighted standardization step exactly as specified here.

#### 2.7.3. DeepMed

DeepMed estimates natural effects by cross-fitting neural-network nuisance functions into the efficient influence functions. We used the deep-neural-network estimator with two hidden layers of 20 units, an L_1_ penalty tuned over {0, 0.05}, 50 training epochs, batch size 100, and propensity trimming at 0.05. Identical hyperparameters, cross-fitting folds, and random seeds were used for both binarization arms described in Section 2.4, so that the two arms differed only in how the binary treatment was constructed and any difference between them was attributable to the exposure contrast rather than to tuning [24].

DeepMed reports five effect quantities: a total effect, direct effects evaluated at the treated and control mediator levels, and indirect effects evaluated at the treated and control exposure levels. Only two of these correspond to the estimands defined in Section 2.4. The natural direct effect as defined there fixes the mediator at its reference-level distribution and therefore corresponds to DeepMed’s control-level direct effect; the natural indirect effect contrasts mediator distributions at the comparative exposure level and corresponds to its treated-level indirect effect. These two, and not the alternative pair, sum to the total effect under the present decomposition, and we extracted this pair for all comparisons. The distinction is immaterial when there is no exposure–mediator interaction, since the two direct effects and the two indirect effects then coincide, but it is not immaterial in the interaction-present scenarios that constitute the more demanding half of the design.

BKMR-CMA completed 100 of 100 replicates in sixteen cells and 97 of 100 in scenario 10; BART-CMA completed 100 of 100 in all seventeen. DeepMed completed 1199 of 1200 fits under the median-split binarization and 1198 of 1200 under the quartile binarization. Uncertainty intervals were the 95% posterior credible interval for the two Bayesian methods and the 95% confidence interval for DeepMed. We report coverage of these native intervals and interpret it as a calibration diagnostic rather than as a claim that Bayesian intervals are constructed to attain frequentist coverage.

### 2.8. Performance Measures

For each scenario, method, and estimand, we report bias, root mean squared error, Monte Carlo standard error, coverage of the nominal 95% interval, mean interval width, and mean runtime. For the plasmode cells, we also report the proportion of replications whose 95% interval excluded zero; where the true effect is non-zero, this is the empirical power to detect it, and where the effect is null, it is the false-positive rate [25]. Convergence and estimation failures were recorded rather than discarded. For each cell, we report the number of successful fits out of 100, and estimates are summarized only over successful fits; a cell with fewer than 10 successful fits was flagged as non-estimable rather than summarized, because performance measures computed from fewer than 10 replicates are not interpretable. Two categories of results were pre-specified for separate reporting: DeepMed estimates in continuous-outcome scenarios, which are excluded from the primary comparison on estimand grounds (Section 2.4), and the binary-outcome scenarios, in which each method met a distinct difficulty. These difficulties are not equivalent and we distinguish them. BKMR-CMA required componentwise variable selection to be disabled because the probit kernel matrix was not positive definite, an implementation constraint rather than an estimation failure, and it went on to produce small bias and near-nominal coverage in both binary cells. BART-CMA produced narrow intervals with coverage of 0.43 and 0.18, which is an estimation failure. DeepMed targets a different estimand throughout. Both categories are reported in full so that the negative results remain on record.

With 100 replications, the Monte Carlo standard error for coverage near 95% is 2.2 percentage points, and the Monte Carlo standard error for bias is one-tenth of the empirical standard error. We report these alongside the corresponding estimates so that differences between methods can be assessed relative to simulation error [25].

### 2.9. Computing Environment and Reproducibility

All simulations were run in R 4.5.0 [40] on the Longleaf high-performance computing cluster (University of North Carolina at Chapel Hill), parallelized as SLURM job arrays with one array task per scenario and shared-memory parallelism across replications within a task. The master seed was 2026; replicate seeds were derived deterministically from the scenario and replicate indices.

### 2.10. Applied Study

#### 2.10.1. Study Population

We conducted an applied mediation analysis among adults in the National Health and Nutrition Examination Survey (NHANES) 2017–2018 cycle, a nationally representative cross-sectional sample of the US civilian non-institutionalized population. Eligibility was defined by membership in the PFAS subsample, a one-third subsample of examined participants aged 12 years or older selected for serum PFAS measurement, rather than by complete PFAS results; subsample members without a PFOA or PFOS value were retained and their values imputed (Section 2.10.5). Of the 1819 subsample members with a complete transient elastography examination, we restricted the analysis to the 1504 aged 20 years or older, because NHANES asks the education question only of participants aged 20 years or older and the smoking and alcohol questions only of those aged 18 years or older; including younger participants would have required imputing answers to questions they were never asked. We then excluded 6 adults whose PFAS subsample weight was zero (coded by NHANES as having no laboratory result), giving an analytic sample of 1498. The analytic dataset comprised 33 variables before imputation, including mixture components, liver outcomes, dietary and cardiometabolic covariates, and NHANES survey design variables (primary sampling unit, stratum, and PFAS subsample weights). The CDC NHANES website provides detailed information on the study design, data collection procedures, and methodologies.

#### 2.10.2. Exposure Mixture

We defined the exposure mixture as five log-transformed biomarkers: perfluorooctanoic acid (PFOA) and perfluorooctane sulfonate (PFOS) measured in serum, and lead, cadmium, and mercury measured in whole blood by inductively coupled plasma mass spectrometry following a simple dilution sample preparation step (ICP-MS; CDC method no. ITB0001A). The blood metals were analyzed using a PerkinElmer NexION 300D ICP-MS (PerkinElmer, Inc., Waltham, MA, USA). To reduce skewness and allow a common shift contrast across components, we transformed each biomarker as log (x + 10^−6^), yielding components {log lead, log cadmium, log mercury, log PFOA, log PFOS}. The causal estimands were defined for a simultaneous shift of each log component from its marginal 25th percentile to its marginal 75th percentile (the Q_25_ → Q_75_ mixture contrast), following the mixture mediation framework used in the simulation study.

#### 2.10.3. Mediator and Outcomes

The mediator M was the Dietary Inflammatory Index (DII), computed from 24 h recall data validated by the Nutrition Methodology Working Group as a continuous summary of the inflammatory potential of the overall diet. The full index draws on 45 dietary components; NHANES 2017–2018 supports 27 of these. Key nutrients used in the DII calculation include carbohydrate, protein, fat, cholesterol, vitamins, minerals, and other dietary elements such as thiamine, riboflavin, beta-carotene, and folic acid. DII scores were calculated separately for each available 24 h recall and averaged, as in our previous analyses of this NHANES cycle [41,42]; participants who completed only the first-day recall contributed that recall alone. Previous studies have validated DII calculations when fewer dietary components are available. Higher DII values indicate more pro-inflammatory diets [43,44]. We considered six liver-related outcomes Y: liver fibrosis (stiffness, kPa) and liver fat (controlled attenuation parameter, dB/m), measured by transient elastography, and four serum liver enzymes (ALT, AST, alkaline phosphatase and GGT), all treated as continuous. For the mediation analysis, outcomes were analyzed on the log scale to stabilize variance and facilitate additive effect contrasts.

#### 2.10.4. Covariates and Confounder Sets

Based on the prior NHANES liver and PFAS/metal literature, we specified a primary confounder set consisting of age, body mass index (BMI), sex, race and ethnicity, education, household income, cigarette smoking, and alcohol use. We included these covariates in both the exposure–mediator and mediator–outcome models and treated them as baseline confounders of the Z–M, M–Y, and Z–Y relationships under the usual natural effect assumptions.

To evaluate sensitivity to potential over-adjustment on downstream cardiometabolic pathways, we fitted a second set of models that additionally adjusted for waist circumference, C-reactive protein (CRP), HbA1c, total and HDL cholesterol, and systolic and diastolic blood pressure. These variables are plausibly influenced by both DII and the exposure mixture and may lie on the diet–inflammation–liver pathway; we therefore interpret this expanded set as a sensitivity analysis rather than as the primary causal adjustment set.

#### 2.10.5. Missing Data and Multiple Imputation

Missing data were addressed using multiple imputation by chained equations (MICE), assuming that values were missing at random given the variables included in the imputation models. This reduces the potential bias and loss of precision associated with complete-case analysis and accounts for uncertainty due to missing data under the missing-at-random assumption.

Missingness was observed in several exposures, covariates, and liver biomarkers; 397 participants (26.5%) had at least one missing value. The largest proportion occurred for household income (133 participants; 8.9%), followed by DII (120; 8.0%), PFOA and PFOS (106 each; 7.1%), AST (93; 6.2%), CRP (90; 6.0%), ALT, alkaline phosphatase and GGT (89 each; 5.9%), total and HDL cholesterol (78 each; 5.2%), alcohol use (70; 4.7%), systolic and diastolic blood pressure (68 each; 4.5%), waist circumference (59; 3.9%), lead, cadmium, and mercury (55 each; 3.7%), HbA1c (54; 3.6%), BMI (12; 0.8%), and education (3; 0.2%). Age, sex, race and ethnicity, smoking status, liver fibrosis, liver fat, and the NHANES survey design variables were complete.

Before imputation, NHANES nonresponse codes were recoded as missing. Education codes for refused and don’t know were set to missing, as were the corresponding income nonresponse codes; valid income and race and ethnicity categories were retained. We specified categorical variables, including sex, race and ethnicity, education, income, smoking, and alcohol use, as factors. Concentrations of lead, cadmium, mercury, PFOA and PFOS were log-transformed prior to imputation.

Twenty imputed datasets were generated using the mice package (version 3.16.0) in R (version 4.3.3) with 20 iterations per dataset and a fixed seed. We used predictive mean matching for continuous variables, including DII, anthropometric and cardiometabolic measures, liver enzyme outcomes, and log-transformed exposures. Logistic regression was specified for binary variables and polytomous regression for multicategory variables; in the adult sample, the only incomplete binary variable was alcohol use, and the incomplete multicategory variables were education and income. All available analysis variables were included in the chained-equation predictor matrix to support compatible and information-rich imputations. Survey design variables such as primary sampling unit, stratum, interview weight, examination weight, and PFAS subsample weight were included as predictors in the imputation models but were not imputed. The observed and imputed distributions showed adequate overlap, supporting the plausibility of the imputations.

#### 2.10.6. Causal Mediation Modelling

For the applied mediation analysis, we used BART-CMA as the primary estimator, selected on the basis of its performance in the plasmode arm, whose support geometry matches that of the applied data. In each imputed dataset, we specified a BART mediator model M | Z, C with 200 trees, and a BART outcome model Y | Z, M, C, using diffuse regularization priors and 2000 burn-in iterations followed by 2000 post-burn-in iterations per model, and thinned posterior samples for the mediation g-computation step. Continuous outcomes were modelled with Gaussian BART; for completeness we also fitted probit BART for binary liver outcomes in exploratory analyses, mapping latent predictions to the probability scale before computing effect contrasts.

Natural direct, indirect, and total effects under the Q_25_ → Q_75_ mixture contrast were obtained by the Bayesian g-formula: for each retained posterior draw, we simulated mediator values under $Z=z_{0}$ and $Z=z_{1}$, propagated them through the outcome model to obtain $Y\left(z_{0},M\left(z_{0}\right)\right),Y\left(z_{1},M\left(z_{0}\right)\right)$, and $Y\left(z_{1},M\left(z_{1}\right)\right)$, and averaged counterfactual outcomes over the empirical confounder distribution in that dataset. The mediator and outcome models were fitted without survey weights, conditional on the confounders, which include the characteristics on which NHANES oversamples (race and Hispanic origin, income, and age). The PFAS subsample weights entered at the standardization step: counterfactual outcomes were averaged over the weighted empirical confounder distribution, so that the natural effects refer to the population represented by the PFAS subsample rather than to the analytic sample. This relies on the fitted models being correct conditional on the confounders, so that the sampling design is ignorable given them. Posterior intervals do not account for the clustering and stratification of the NHANES design and may therefore be somewhat too narrow. We stacked posterior draws of the TE, NDE, and NIE across the 20 imputations and treated them as arising from a mixture of posteriors; point estimates were the pooled posterior means, and 95% credible intervals were the central 95% of pooled draws. For each outcome, we also computed the proportion mediated (PM = NIE/TE), recognizing that PM is unstable when TE is near zero, and therefore reporting it primarily as a descriptive quantity.

#### 2.10.7. Sensitivity Analyses

We repeated the BART-CMA mediation analysis under the expanded confounder set that included waist circumference, CRP, HbA1c, lipids, and blood pressure, using identical exposure, mediator, outcome, and weighting specifications. Comparing estimates across the primary and expanded sets allowed us to assess the impact of adjusting for potential downstream cardiometabolic variables on the NIE and TE, and to illustrate the consequences of over-adjustment in diet–inflammation mediation models within the NHANES mixtures setting.

## 3. Results

### 3.1. Synthetic Simulation

Table 2 summarizes NIE performance across the seventeen synthetic scenarios, and Figure 3 displays the results. Results for the TE and NDE are given in Supplementary Table S1.

#### 3.1.1. Bias

NIE bias differed substantially between BART-CMA and BKMR-CMA across the synthetic conditions (Figure 3a). In scenarios without an exposure–mediator interaction (scenarios 1, 2, 5 and 6), BART-CMA was approximately unbiased, with bias ranging from −0.031 to −0.002, whereas BKMR-CMA showed modest positive bias ranging from 0.119 to 0.203. When exposure–mediator interactions were introduced, BART-CMA exhibited much larger bias than BKMR-CMA. Under a linear data-generating mechanism (scenarios 3 and 4) BART-CMA underestimated the NIE, with bias of −0.665 at *n* = 300 and −0.483 at *n* = 1000, whereas BKMR-CMA remained closer to zero (0.041 and 0.077). A similar pattern was observed under nonlinear exposure–response relationships (scenarios 7 and 8): BART-CMA bias was −0.696 at *n* = 300 and −0.436 at *n* = 1000, compared with −0.009 and 0.006 for BKMR-CMA. Increasing the sample size therefore reduced the magnitude of BART-CMA bias in the continuous-outcome interaction settings but did not eliminate it.

For the binary-outcome scenarios (9 and 10), both methods exhibited negative bias, although the magnitude was smaller for BKMR-CMA: BART-CMA bias was −0.119 and −0.116 at *n* = 300 and *n* = 1000, compared with −0.050 and −0.040 for BKMR-CMA. The largest divergence between methods occurred when the number of mixture components carrying effects was varied. With three mixture components (scenario 11), BART-CMA underestimated the NIE by −0.495, compared with −0.038 for BKMR-CMA, and with ten components carrying effects (scenario 12), BART-CMA bias increased to −2.369, whereas BKMR-CMA remained close to zero at −0.058. Overall, BART-CMA performed well without exposure–mediator interaction but showed increasing negative bias under exposure–mediator interaction and binary outcomes, and when more mixture components carried effects, whereas BKMR-CMA showed modest bias in the simpler no-interaction settings but was comparatively more stable under the more complex conditions.

With the signal held fixed (scenarios 15–16), so that the true effects equaled those of scenario 11, the NIE bias of BART-CMA was essentially unchanged as the mixture grew (−0.495, −0.448, and −0.508 at L = 3, 5, and 10; relative bias −32%, −29%, and −33%; Table 2), and BKMR-CMA remained close to the truth (relative bias −2%, −3%, and −2%). By contrast, in scenario 12, where the seven additional exposures carried nonzero mediator, outcome, and interaction coefficients and the true NIE was three times larger, BART-CMA’s relative bias was −51%. The same pattern held for the total and natural direct effects (Supplementary Table S1): BART-CMA recovered the total effect in all four scenarios (relative bias −2% to +4%), and the indirect effect it failed to capture reappeared in its direct-effect estimate (NDE bias +0.47, +0.55, and +0.46 at L = 3, 5, and 10, and +2.37 in scenario 12). For both estimators, adding inert exposures increased the empirical standard error of each effect by 26–42% between L = 3 and L = 10 (for the NIE, from 0.199 to 0.261 for BART-CMA and from 0.186 to 0.257 for BKMR-CMA) without increasing bias.

Raising the exposure correlation, imposing a block correlation structure, or doubling the nonlinear terms changed little (scenarios 17–19). Relative to scenario 7, the matched baseline, BKMR-CMA NIE bias moved from −0.009 to −0.062 under an exchangeable correlation of 0.7, −0.052 under the block structure, and +0.021 under doubled nonlinearity, while BART-CMA bias moved from −0.696 to −0.746, −0.733, and −0.658. Coverage followed the same ordering, 0.97 to 0.99 for BKMR-CMA against 0.52 to 0.64 for BART-CMA. The total and natural direct effects behaved as in the other interaction-present scenarios (Supplementary Table S1): BART-CMA recovered the total effect in all three cells (relative bias −3% to +2%) while the indirect effect it failed to capture reappeared in its direct-effect estimate (NDE bias +0.71, +0.64 and +0.73). Stronger or structured exposure correlation and stronger nonlinearity therefore did not alter the relative standing of the two estimators within this condition.

#### 3.1.2. Root Mean Squared Error

RMSE for the NIE varied across simulation settings, with clear contrasts between BART-CMA and BKMR-CMA (Figure 3b). In the linear scenarios without an exposure–mediator interaction (scenarios 1 and 2), BART-CMA had slightly lower RMSE than BKMR-CMA: at *n* = 300, RMSE was 0.242 for BART-CMA and 0.263 for BKMR-CMA, decreasing at *n* = 1000 to 0.151 and 0.169, respectively.

When an exposure–mediator interaction was introduced under a linear mechanism (scenarios 3 and 4), this pattern reversed: at *n* = 300, RMSE was 0.709 for BART-CMA and 0.225 for BKMR-CMA, and although RMSE decreased for both methods at *n* = 1000 (0.542 versus 0.159) a substantial gap remained. Under nonlinear exposure–response relationships without interaction (scenarios 5 and 6), the methods performed more similarly: at *n* = 300, BART-CMA had lower RMSE than BKMR-CMA (0.219 versus 0.280), whereas at *n* = 1000, the values were nearly identical (0.174 versus 0.168). When nonlinearity and exposure–mediator interaction were both present (scenarios 7 and 8), BKMR-CMA clearly outperformed BART-CMA: at *n* = 300, RMSE was 0.760 for BART-CMA and 0.259 for BKMR-CMA, improving to 0.514 and 0.150, respectively, at *n* = 1000.

For the binary-outcome scenarios (9 and 10), overall RMSE was relatively low for both methods, but the comparison consistently favored BKMR-CMA: at *n* = 300, RMSE was 0.122 for BART-CMA and 0.076 for BKMR-CMA, and at *n* = 1000, it was 0.120 versus 0.057. The number of components carrying effects had the largest effect on estimation error. With three mixture components (scenario 11), RMSE was 0.533 for BART-CMA and 0.189 for BKMR-CMA, and increasing to ten components carrying effects (scenario 12) substantially inflated RMSE for both methods, to 2.438 for BART-CMA and 0.674 for BKMR-CMA. With ten components but the signal held fixed (scenario 16), RMSE was 0.570 and 0.258, respectively. Overall, although larger sample sizes reduced RMSE, BART-CMA remained particularly sensitive to exposure–mediator interaction and to additional carrying effects, whereas BKMR-CMA achieved lower RMSE in most of the more complex settings.

#### 3.1.3. Coverage Probability

Coverage of the 95% intervals for the NIE differed sharply between the two methods (Figure 3c). BKMR-CMA maintained coverage at or very near the nominal 0.95 level across all seventeen scenarios, regardless of linearity, exposure–mediator interaction, sample size, outcome type, or mixture dimensionality. BART-CMA achieved near-nominal coverage only in scenarios without exposure–mediator interaction (0.97 to 0.99 in the linear and nonlinear no-interaction settings). Coverage dropped markedly when interactions, binary outcomes, or additional components carrying effects were introduced: to 0.60 to 0.71 under interaction, to 0.43 and 0.18 in the binary-outcome scenarios, and to 0.66 and 0.13 with three and ten mixture carrying effects, respectively (0.84 and 0.81 at L = 5 and L = 10 with the signal held fixed), and to 0.52, 0.57, and 0.64 under stronger or structured exposure correlation and stronger nonlinearity (scenarios 17–19). BKMR-CMA therefore delivered well-calibrated NIE intervals across the synthetic settings, whereas BART-CMA showed pronounced undercoverage outside the simpler continuous, no-interaction scenarios.

#### 3.1.4. Interval Width

Interval widths for the NIE were generally narrower for BKMR-CMA than for BART-CMA across most continuous-outcome scenarios (Figure 3d). In both linear and nonlinear settings without exposure–mediator interaction, BKMR-CMA produced slightly shorter intervals than BART-CMA at each sample size, and these widths decreased as n increased. When exposure–mediator interaction was introduced, either alone or combined with nonlinearity, differences became more pronounced, with BKMR-CMA consistently yielding shorter intervals even as both methods benefited from larger sample sizes. In the binary-outcome scenarios, BART-CMA produced narrower intervals than BKMR-CMA, but this apparent gain in precision coincided with severe undercoverage, indicating overconfident and poorly calibrated intervals rather than improved inference.

Interval widths increased for both methods from three to ten components carrying effects (scenarios 11 and 12), but BKMR-CMA retained narrower intervals than BART-CMA while still maintaining near-nominal coverage. With the signal held fixed (scenarios 15–16), BART-CMA intervals widened modestly (from 1.175 at L = 3 to 1.503 at L = 10), whereas BKMR-CMA intervals were essentially unchanged (1.035–1.088). Overall, BKMR-CMA typically delivered shorter, well-calibrated intervals, whereas BART-CMA produced narrower intervals primarily when coverage fell well below the 0.95 target.

#### 3.1.5. Runtime per Replication

Runtime per replication varied markedly across methods, with BKMR-CMA generally much faster than BART-CMA in most synthetic scenarios (Figure 3e). In the core continuous-outcome settings, BART-CMA runtimes were typically of the order of 10^3^ to 10^4^ s per replication, whereas BKMR-CMA ranged from about 10^2^ to 10^3^ s. Runtime increased with sample size for both methods and, to a lesser extent, with mixture size for BART-CMA. Across the linear and nonlinear continuous-outcome scenarios, increasing *n* from 300 to 1000 multiplied BKMR-CMA runtimes by about 5 to 13 and BART-CMA runtimes by about 3 to 6, with BKMR-CMA remaining consistently faster in all but one setting. Mixture size also added computational burden for BART-CMA: at *n* = 300, increasing from three to ten components raised BART-CMA runtime from about 1500 to 2800 s, and to about 2500 s when the seven added components were inert (scenario 16), whereas BKMR-CMA stayed at about 160 to 210 s. In scenarios 17–19, BKMR-CMA required about 90 s per replication and BART-CMA 1350 to 2257 s.

The main exception was the binary-outcome scenario at *n* = 1000, where BKMR-CMA runtime exceeded 13,000 s, longer than that of BART-CMA (about 7900 s). Overall, BKMR-CMA was more computationally efficient than BART-CMA in 16 of the 17 scenarios while still delivering lower or comparable RMSE and better-calibrated intervals in most settings.

#### 3.1.6. Sensitivity to BKMR-CMA Variable Selection

When variable selection was disabled in both arms, BKMR-CMA exhibited minimal bias for the natural indirect effect (NIE) in continuous scenarios 7 and 8 (bias values of −0.004 and −0.001; relative bias less than 1%). In contrast, in binary scenarios 9 and 10, the NIE bias was −0.050 and −0.040 (relative bias −26% and −20%). This difference between continuous and binary arms is attributable to the outcome type rather than to changes in model specification. Using identical data, disabling variable selection altered the NIE bias by no more than 0.025 (scenarios 1, 7, and 8) and changed the mean interval width by at most 4%. The NDE bias was marginally smaller without variable selection (−0.060 vs. +0.018, −0.057 vs. +0.025, and −0.113 vs. −0.062 in scenarios 1, 7, and 8, respectively; Supplementary Table S3).

### 3.2. Plasmode Simulation

Table 3 summarizes performance in the two NHANES-based plasmode cells, and Figure 4 shows it.

Bias of the NIE was minimal for both methods under the null scenario (scenario 13), where BART-CMA and BKMR-CMA each produced NIE estimates essentially equal to zero under the NHANES-like exposure and confounder structure (Figure 4a). In the non-null scenario (scenario 14), performance diverged: against a true NIE of −0.0394, BKMR-CMA had a bias of +0.0725, corresponding to a mean estimate of +0.033, so the mediated pathway was not merely attenuated towards the null but estimated with the wrong sign, whereas BART-CMA had a bias of +0.0036 and remained close to the true value. Thus, while both approaches recovered the null NIE in scenario 13, BART-CMA had substantially lower bias than BKMR-CMA when the mediated pathway was strengthened. Neither method, however, reliably distinguished the imposed effect from zero. The 95% interval excluded zero in 1% of BART-CMA replications and in none of the BKMR-CMA replications (Figure 4e; Table 3). For the NDE in scenario 14, BKMR-CMA intervals excluded zero in 22% of replications, but with a bias of −0.0992 and coverage of 0.92, this reflects estimates displaced from the truth rather than accurate detection.

RMSE for the NIE was low for both methods under the null plasmode scenario, with BART-CMA showing smaller error than BKMR-CMA (0.0017 versus 0.0083), indicating that both approaches recovered the effectively null mediated effect with minimal estimation error (Figure 4b). When a non-null NIE was imposed, RMSE increased for both methods but remained lower for BART-CMA (0.1777) than for BKMR-CMA (0.2471), consistent with the larger bias observed for BKMR-CMA in this setting. BART-CMA therefore exhibited lower RMSE in both plasmode scenarios, with the performance gap widening when the mediated pathway was strengthened.

Coverage of the 95% intervals for the NIE was high for both methods in the plasmode simulations (Figure 4c). Under the null scenario, both methods achieved coverage of 1.00, exceeding the nominal 0.95 target. When a non-null NIE was introduced, coverage remained at 1.00 for BKMR-CMA and 0.99 for BART-CMA. High coverage in scenario 14 reflects wide intervals rather than precise ones: the mean interval was about 22 times the size of the true NIE for BART-CMA and about 39 times for BKMR-CMA, so almost every interval contained zero as well as the true value.

Interval widths for the NIE differed between methods (Figure 4d). Under the null scenario, BART-CMA produced much narrower intervals than BKMR-CMA (0.0176 versus 0.0848). When a non-null NIE was introduced, widths increased for both methods but remained smaller for BART-CMA (0.8794) than for BKMR-CMA (1.5243). In the NHANES-based plasmode setting, BART-CMA therefore yielded narrower intervals than BKMR-CMA, although even these intervals were far too wide to exclude zero.

### 3.3. DeepMed Performance

DeepMed results are reported separately from the primary comparison because the binarized treatment does not represent the componentwise Q_25_ → Q_75_ contrast (Section 2.4). Against the target truth, DeepMed’s NIE estimates were negative in all eight core scenarios under both binarizations, whereas the true NIE was positive in every scenario; in scenarios 9–12, the estimates were mostly positive but far below the truth (for example, 0.105 and 0.494 against 4.633 in scenario 12). Absolute relative bias in the NIE ranged from 57% to 153%, and the 95% confidence interval covered zero in eleven of the twelve scenarios under both binarizations, reaching at most 0.12 in scenario 10. Scoring DeepMed against the truth for its own binary contrast showed that little of this error is attributable to the estimand mismatch (Supplementary Table S4). The median split implies a narrower contrast than the target (mean componentwise shift 1.11 versus 1.38 in the five-exposure scenarios), so its own-contrast truth is smaller; the mismatch accounted for 14% to 35% of the NIE bias, and the remainder was estimation error. The quartile binarization implies a wider contrast (1.77), so its own-contrast truth is larger than the target, and the mismatch offsets rather than explains the bias: against its own-contrast truth, DeepMed’s NIE bias was 1.1 to 1.9 times larger than against the target. Coverage of the own-contrast truth was zero in eleven of the twelve scenarios under both binarizations, and the own-contrast truths were positive in every scenario, so the negative estimates cannot be explained by the contrast change. The same pattern was present, though less extreme, in the TE and NDE against the target truth. Supplementary Figures S1–S6 and Supplementary Tables S2 and S4 show these results. Because the estimates fail against both truths, they reflect how DeepMed performed in this configuration, in which the treatment was derived from the exposure vector that was also included among the covariates (Section 2.4), rather than from the change in the estimand alone.

### 3.4. Applied NHANES Analysis

#### 3.4.1. Correlation Structure

Figure 5 presents the Spearman correlation matrix for the environmental mixture components, DII, and the six liver outcomes. Correlation analysis demonstrated modest dependence among components of the environmental mixture, with the strongest exposure correlation observed between PFOA and PFOS (ρ = 0.59), followed by lead and cadmium (ρ = 0.34) and mercury and PFOS (ρ = 0.28). Lead was also positively correlated with PFOS (ρ = 0.23) and PFOA (ρ = 0.19). Correlations between individual exposures and DII were weak, ranging from −0.14 for mercury to 0.12 for cadmium. DII was also weakly correlated with the liver outcomes: liver fibrosis (ρ = 0.02), liver fat (ρ = 0.02), ALT (ρ = −0.12), AST (ρ = −0.12), alkaline phosphatase (ρ = 0.08), and GGT (ρ = −0.03). Stronger correlations were observed among the liver biomarkers themselves, particularly ALT and AST (ρ = 0.73), ALT and GGT (ρ = 0.54), and AST and GGT (ρ = 0.40). Liver fat was positively correlated with liver fibrosis (ρ = 0.32), ALT (ρ = 0.31), and GGT (ρ = 0.29). The correlation structure therefore showed appreciable dependence within the environmental mixture and among the liver outcomes, but comparatively weak pairwise relationships involving DII. The corresponding matrix extended to the primary confounder covariates is given in Supplementary Figure S7.

#### 3.4.2. Natural Indirect Effect Through DII

In a BART-CMA analysis of 1498 adults, standardized to the PFAS subsample weights and pooling posterior draws across the 20 multiply imputed NHANES datasets, we evaluated whether DII mediated associations between a joint mixture of lead, cadmium, mercury, PFOA and PFOS, and the six liver outcomes. The exposure contrast compared the joint mixture at the 75th percentile with that at the 25th percentile, and all liver outcomes were modeled on the log scale. Full estimates for the TE, NDE, NIE, and proportion mediated are given in Table 4.

The estimated natural indirect effects through DII were close to the null for all six liver outcomes (Figure 6b). For liver fibrosis, the NIE was −0.0027 (95% credible interval −0.0127, 0.0043), and for liver fat, it was −0.0010 (−0.0059, 0.0022). The NIEs for alkaline phosphatase and GGT were −0.0014 (−0.0085, 0.0033) and −0.0033 (−0.0180, 0.0060), respectively. We observed small positive point estimates for ALT (0.0028; −0.0047, 0.0145) and AST (0.0023; −0.0035, 0.0112).

All 95% intervals included zero, providing no clear evidence that DII mediated the association between the exposure mixture and any liver outcome. On the log outcome scale, the NIE point estimates corresponded to changes of less than approximately 0.4% in each liver outcome through the DII pathway. Although the point estimates for ALT and AST were positive, whereas those for liver fibrosis, liver fat, alkaline phosphatase and GGT were negative or near zero, the intervals were compatible with small indirect effects in either direction.

#### 3.4.3. Total Effect of the Mixture on Liver Markers

Figure 6a presents the estimated total effects of a joint increase in the lead, cadmium, mercury, PFOA, and PFOS mixture from the 25th to the 75th percentile. Total-effect point estimates were negative for all six liver outcomes, although their precision varied considerably and the estimate for GGT was essentially zero. The strongest association was observed for liver fibrosis, with a total effect of −0.1986 (95% credible interval −0.3416, −0.0642). Because liver fibrosis was modeled on the log scale, this corresponds to an approximately 18.0% lower liver fibrosis value for the higher-versus-lower mixture contrast, calculated as 100 × [exp(−0.1986) − 1]; the interval excluded zero.

For liver fat the estimated total effect was also negative at −0.0447 (−0.1163, 0.0224), corresponding to an approximately 4.4% lower value on the original scale, although the interval included zero. The remaining liver markers also had inverse point estimates: ALT, −0.0572 (−0.2372, 0.1134); alkaline phosphatase, −0.0442 (−0.1589, 0.0729); AST, −0.0139 (−0.1388, 0.1049); and GGT, −0.0019 (−0.2400, 0.2210). For each outcome, the interval spanned zero and was compatible with inverse, null, or positive associations. The applied analysis therefore showed a consistent pattern of inverse total-effect point estimates across liver outcomes, but liver fibrosis was the only outcome for which the 95% interval excluded zero.

#### 3.4.4. Proportion Mediated Through DII

We calculated the proportion mediated through DII for each pooled posterior draw as the natural indirect effect divided by the total effect, PM = NIE/TE. Posterior draws from the 20 multiply imputed datasets were concatenated, and the reported point estimates and 95% intervals summarize the resulting distribution of draw-specific estimates (Figure 6c).

The estimated proportion mediated varied across the six liver outcomes. The largest positive point estimate was observed for GGT (38.1%), followed by liver fat (4.4%), liver fibrosis (1.7%), and ALT (0.7%); the estimates for alkaline phosphatase (−0.1%) and AST (−3.7%) were negative. These estimates were highly uncertain, and the 95% interval included zero for every outcome: liver fibrosis, −2.6% to 9.0%; liver fat, −25.7% to 39.6%; ALT, −71.5% to 60.1%; AST, −81.7% to 87.1%; alkaline phosphatase, −46.2% to 46.7%; and GGT, −65.6% to 77.1%. The relatively large point estimate for GGT should be interpreted cautiously because its total effect (−0.0019) and natural indirect effect (−0.0033) were both small and imprecisely estimated. When the total effect is close to zero, the ratio NIE/TE becomes unstable, producing highly variable proportion-mediated estimates; the posterior standard deviation of the proportion mediated (Table 4) reaches 22.1 for GGT, and for several outcomes, the posterior mean of the ratio differs in sign or size from the ratio of the NIE and TE point estimates, which is itself a signature of a heavy-tailed ratio rather than a computational error.

Overall, the proportion-mediated estimates provide no clear evidence that DII accounted for a substantial or precisely estimated fraction of the mixture associations with liver outcomes. The small proportion mediated for liver fibrosis is consistent with its near-null NIE. The negative estimate for AST reflects the opposite signs of the TE and NIE point estimates together with substantial uncertainty, and should not be interpreted as evidence of a protective mediated pathway.

#### 3.4.5. Over-Adjustment Sensitivity Analysis

In the over-adjustment sensitivity analysis, we additionally adjusted for variables potentially downstream of the exposure mixture or DII (Table 5). Natural indirect effects remained close to zero and imprecise across all six liver outcomes (Figure 7b). The largest positive NIEs were observed for ALT (0.0028; −0.0043, 0.0142) and AST (0.0019; −0.0037, 0.0095). NIE estimates were also close to the null for liver fibrosis (−0.0009; −0.0070, 0.0033), liver fat (−0.0006; −0.0043, 0.0021), GGT (−0.0008; −0.0100, 0.0072), and alkaline phosphatase (0.0000; −0.0043, 0.0041). All intervals included zero, providing little evidence that DII mediated associations between the exposure mixture and the evaluated liver outcomes. The persistence of near-null NIE estimates after expanded adjustment indicates that the overall conclusion was not materially changed. However, because the additional covariates may be downstream of the exposure mixture or DII, we interpret these results as a sensitivity analysis rather than primary causal mediation estimates.

Total-effect point estimates were negative for five of the six liver outcomes (Figure 7a). Liver fibrosis had the strongest inverse association (−0.1320; −0.2582, −0.0143), about one-third smaller than in the primary analysis (−0.1986). Liver fat was also inversely estimated (−0.0327; −0.0935, 0.0264), but its interval included zero. ALT (−0.0404; −0.1973, 0.1171), alkaline phosphatase (−0.0237; −0.1349, 0.0782), and AST (−0.0098; −0.1214, 0.1008) had small inverse point estimates, whereas the point estimate for GGT was positive (0.0332; −0.1649, 0.2226); all four intervals included zero. Only the inverse association with liver fibrosis remained distinguishable from the null under the expanded adjustment.

Estimated proportions mediated were unstable for several liver outcomes because the corresponding total effects were small and imprecisely estimated (Figure 7c). The largest positive point estimates were for ALT (43.2%) and AST (6.8%); estimates were 1.8% for liver fibrosis and liver fat, 0.6% for alkaline phosphatase, and −4.4% for GGT, and every 95% interval included zero. These percentages should not be interpreted as literal fractions of the mixture effect operating through DII. The ALT estimate results from dividing a near-null indirect effect (0.0028; −0.0043, 0.0142) by a small, uncertain total effect, indicating an unstable ratio rather than substantial mediation. Overall, the sensitivity analysis provided little evidence that DII accounted for a substantial proportion of the mixture–liver associations.

## 4. Discussion

This study compared flexible approaches for causal mediation analysis of correlated environmental exposure mixtures across controlled synthetic simulations, an NHANES-based plasmode simulation, and an applied analysis of a joint PFAS–metal mixture, the Dietary Inflammatory Index (DII), and liver-related outcomes. The primary comparison focused on BKMR-CMA and BART-CMA, both of which can target a prespecified intervention on a multivariate continuous exposure vector; we evaluated DeepMed separately because its binary treatment representation does not target the same continuous mixture estimand. No single method is superior for estimating mediation in a correlated PFAS-metal mixture; which estimator performed better largely depends on the data structure in which it was applied. In NHANES, diet-related inflammation, operationalized by DII, mediated little of the mixture’s association with liver biomarkers, offering little support for the inflammatory pathway proposed to drive environmentally induced hepatotoxicity. Comparative performance depended on the target estimand, the exposure contrast, the outcome scale, exposure–mediator interaction, the number of mixture components carrying effects, the empirical support available for cross-world counterfactual predictions, and the implementation of posterior uncertainty quantification. These results indicate that the choice of method in environmental-mixture mediation analysis should be driven by the scientific intervention and the anticipated structure of the analytic data rather than by a general ranking of modeling flexibility [14,17,39].

In continuous-outcome simulations without exposure–mediator interaction, BART-CMA showed slightly lower NIE bias and RMSE than BKMR-CMA. This suggests that BART-CMA can accurately estimate mixture-mediated effects when the observed data adequately support nonlinear relationships between the mediator and the outcome. Its tree-based framework flexibly captures nonlinearities and interactions without requiring prespecified functional forms [38,39].

BART-CMA performed worse when an exposure–mediator interaction was present, underestimating the NIE and exhibiting higher bias, RMSE and interval undercoverage, especially with ten mixture components, where its bias was −2.369 against −0.058 for BKMR-CMA. BKMR-CMA generally produced lower errors and near-nominal coverage, likely because its kernel framework more effectively captures complex nonlinear and interactive relationships among correlated exposures. This pattern is consistent with the cross-world nature of natural indirect effects. The NIE compares outcomes under one exposure setting while allowing the mediator to take values it would have had under another, so when exposure–mediator interaction is present, the effect of changing the mediator depends on the exposure level at which the outcome is evaluated. Estimation may therefore require predictions for rarely observed exposure–mediator combinations, making results vulnerable to poor overlap, extrapolation, and model misspecification. In the simulations, strong positive mixture–mediator associations produced sparse cross-world support, creating a deliberate stress test for indirect-effect estimation [26,29,30]. The fixed-signal scenarios show that the complexity in the larger mixture was not the number of components itself but the additional effects those components carried. When the added exposures were inert, neither estimator became more biased, though both lost some precision; when they contributed additional mediator, outcome, and interaction terms, BART-CMA’s relative NIE bias rose from about one-third to one-half of the true effect. Inert exposures represent a sparse setting that componentwise variable selection in BKMR is designed to exploit [35]; BART-CMA, whose default prior selects splitting variables uniformly, was nonetheless not further biased by them [38]. Method selection should therefore consider the expected number of components carrying effects, interaction complexity, computational resources, and application-informed simulations together.

The binary-outcome simulations demonstrated that accuracy and uncertainty calibration require separate assessment. Although both Bayesian methods occasionally achieved low RMSE, BART-CMA often showed undercoverage even when its intervals were narrower than BKMR-CMA’s. Bias, RMSE, interval width, and coverage should therefore be reported together. For binary outcomes, effects should be calculated on the same scale as the simulation reference; in this analysis, probit-model predictions were transformed to the probability scale before estimating natural indirect effects as risk differences [25,31,45].

A key methodological contribution of this study is its emphasis on estimand alignment. For multivariate exposures, there is no single causal parameter that can be described simply as the mixture effect; the intervention on the exposure vector must first be defined. We specified a simultaneous shift in lead, cadmium, mercury, PFOA, and PFOS from the marginal 25th to the 75th percentile. We configured BKMR-CMA and BART-CMA to estimate this componentwise continuous-mixture contrast directly. DeepMed requires a binary treatment, so its median-split and extreme-quartile mixture-index contrasts represented different interventions. An index-based exposure classifies individuals on realized aggregate exposure values, whereas the target intervention sets each component to a specified percentile. People with similar index values can therefore have different PFAS and metal profiles, and binary treatment definitions need not produce the same average componentwise shift as the prespecified mixture intervention.

This mismatch is not merely technical. Dichotomizing a continuous mixture discards information and can reduce power, while adjusting for the full exposure vector after deriving a binary treatment from that same vector can leave little residual treatment variation, creating a practical positivity problem [46]. DeepMed should consequently not be regarded as directly comparable with BKMR-CMA and BART-CMA for the primary continuous-mixture estimand. Scoring DeepMed against the truth for its own binary contrast showed that the change of estimand explained at most about a third of its NIE bias, and none of it under the quartile binarization; most of the error was estimation error, consistent with the positivity problem created when the treatment is derived from an exposure vector that is also adjusted for. Its results therefore illustrate two separate costs of forcing a multivariate continuous exposure mixture into a binary treatment framework: a change of estimand, and a configuration in which the binary-treatment estimator itself performs poorly. Explicitly defining the intervention, potential outcomes, and causal estimand before model fitting is essential for valid comparison of mediation methods [17,29].

The plasmode simulation showed why synthetic findings should be interpreted alongside application-informed evaluation. In the synthetic data, the mixture–mediator relation was strong and positive, with sparse cross-world support. In the observed NHANES structure, the mixture–DII relation was weak and negative, and cross-world support was considerably greater: the mixture index and mediator correlation was approximately −0.06 in NHANES against 0.66 to 0.85 in the synthetic scenarios, and cross-world occupancy was approximately 6.8% against 0.04% to 0.78%. The plasmode retained empirical exposure and confounder dependence while generating mediator and outcome values from known structural equations. This preserves realistic multicollinearity, confounding, and overlap patterns that are difficult to recreate in fully parametric simulations [32,33].

In the NHANES-based plasmode, both methods recovered null NIEs, but when a negative NIE was imposed, BART-CMA had lower bias and RMSE, narrower intervals, and near-nominal coverage, whereas BKMR-CMA returned a positive mean estimate, and so reversed the sign of the mediated pathway. Neither method, however, could distinguish that imposed effect from zero: the interval excluded zero in 1% of BART-CMA replications and in none of the BKMR-CMA replications. Detecting a mediated effect of this size would require samples substantially larger than those studied here, because the intervals both methods produced were an order of magnitude wider than the effect itself. The near-null applied estimates are therefore consistent with little or no mediation through DII, but they cannot rule out a small indirect effect of the size imposed in the plasmode, which neither method detected in samples of 1000 drawn from these data, somewhat fewer than in the applied analysis. Together, these results suggest that estimator performance in mixture mediation depends on both the estimator and the data structure, and they offer little support for DII as a major route linking this mixture to liver outcomes in US adults.

Implementation also affected inference. In BART-CMA, mediator simulations must remain paired with the posterior draws that generated them in order to propagate uncertainty fully. Breaking this pairing left point estimates similar but produced overly narrow intervals; correcting it increased mean NIE interval widths by 55% to 98%. A discrepancy between the published BKMR-CMA algorithm and its software implementation could likewise bias indirect effects towards zero. Both defects produce output that looks substantively plausible, which underscores the need for known-truth simulations, transparent code, and independent implementation checks [25].

In NHANES, DII mediated little of the association between the joint PFAS–metal mixture and any of the six liver outcomes, with estimated mediated changes below 0.4% on the original outcome scale. An indirect effect requires the mixture to shift DII and DII to shift the liver outcome, and neither path was evident. The mixture explained only a few percent of the variance in DII, with component-specific correlations of approximately −0.14 to 0.12, and DII’s correlations with ALT and AST were weakly inverse. This differs from earlier reports, in which higher dietary inflammatory potential was associated with adverse hepatic outcomes: DII correlated positively with ALT and AST, and was associated with NAFLD and advanced fibrosis in NHANES 2005–2016 [47]; it was associated with NAFLD in a meta-analysis [48] and with NAFLD and advanced hepatic fibrosis in other studies [13]; and Ramírez-Vélez et al. reported associations with several hepatic health measures in US adults, although less consistently for transient elastography [49]. Because the DII used here was not energy-adjusted, energy intake and misreporting may contribute to this discordance [50]. The near-null findings therefore do not imply that inflammation is irrelevant to liver health. DII is not a direct measure of systemic or hepatic inflammation, oxidative stress, or toxicant-related metabolic dysfunction, and those studies asked whether DII is associated with liver health, whereas we asked whether it transmits the effect of a defined mixture intervention. A mediator can be associated with an outcome yet transmit little of an exposure’s effect if the exposure barely changes it. Other biologically plausible mediators include oxidative stress biomarkers, insulin resistance, metabolic syndrome markers, and gut microbiome-related measures that may account for portions of the mixture–liver association that DII does not capture.

Several features of the hypothesized pathway may explain why the mixture changed DII so little. Diet is itself a route of PFAS exposure, and DII has been associated with serum PFOA, PFNA, and PFOS concentrations in NHANES [51], so DII may act more as a determinant of exposure than as an intermediate. PFAS and metal hepatotoxicity is attributed largely to direct actions on the liver, through reactive oxygen species, disrupted lipid metabolism, and peroxisome proliferator-activated receptor (PPAR-α) activation [5,9], which would appear in the natural direct effect. Oxidative stress may therefore be a more proximal mediator than dietary inflammation; biomarkers such as F2-isoprostanes, oxidized LDL, glutathione-related measures, or urinary 8-OHdG would provide more direct tests of this pathway than DII. Insulin resistance and metabolic-syndrome markers, including fasting glucose, insulin or HOMA-IR, triglycerides, waist circumference, and adiposity, are also plausible intermediates because PFAS exposure has been linked to altered glucose homeostasis, dyslipidemia, and metabolic dysfunction; triglycerides, for example, have been reported to mediate associations between PFAS mixtures and NAFLD risk scores. Gut-microbiome pathways represent an additional possibility, operating through altered microbial composition, intestinal permeability, microbial metabolites, bile acids, and endotoxin-related inflammatory signaling along the gut–liver axis.

PFASs have even been inversely associated with serum TNFα [52], and where systemic inflammation was tested as a mediator, it carried an estimated 3.5% of a PFOS–NAFLD association [53]. Diet may also modify rather than transmit these effects [47,51,54], and modification does not appear as an indirect effect. Finally, cadmium and mercury were associated with DII in opposite directions in this NHANES cycle [41], and mixture components can act through pathways with opposite associations with disease risk [55], so a joint contrast can let component-specific indirect effects offset one another. Our null finding parallels the limited mediation by allostatic load of associations between metals and liver outcomes in the same cycle [56].

The inverse total effect on liver fibrosis is counterintuitive given prior evidence linking PFOA and PFOS to adverse hepatic outcomes, although associations in NHANES have varied across PFAS compounds and liver phenotypes [57–59]. This pattern may arise because the joint mixture contrast does not isolate the hepatotoxic effects of individual components. In particular, serum PFOA concentrations in cross-sectional data reflect both exposure and elimination, and reduced kidney function can elevate measured serum PFOA independently of recent intake [60]. Thus, the observed contrast may partly reflect differences in kidney function or PFAS elimination rather than a causal protective effect on fibrosis.

The mercury component may also influence the direction of the joint contrast. Blood organic mercury largely reflects fish and shellfish intake, and seafood consumption is correlated with intake of omega-3 and omega-6 fatty acids and with broader dietary patterns that may be associated with lower hepatic risk [61]. Consequently, a contrast that increases mercury may partially capture the health profile of participants with higher seafood consumption rather than mercury’s independent effect on liver fibrosis. Component-specific contrasts and models incorporating kidney function would help clarify whether the inverse total effect reflects a mixture-level association, reverse causation, or dietary pattern.

Interpret total-effect estimates cautiously. Point estimates were below zero for all six outcomes, but only liver fibrosis had an interval excluding zero. The liver-fibrosis total effect of −0.199 on the log scale corresponds to an estimated 18.0% lower fibrosis value under the joint Q_25_ → Q_75_ mixture contrast. This inverse association should not be interpreted as evidence of a protective effect. Existing toxicologic and epidemiologic evidence generally supports adverse hepatic effects of PFAS exposure, particularly for PFOA and PFOS, although associations vary across chemicals, populations, outcomes, and analytic approaches [57]. NHANES studies have reported PFAS associations with fibrosis-related outcomes and heterogeneous associations across PFAS compounds and liver phenotypes [58,59]. A joint mixture intervention need not reproduce the direction or magnitude of single-chemical associations when mixture components are correlated and response surfaces are nonlinear or interactive.

Proportion-mediated estimates were unstable when total effects were small or uncertain. GGT had an estimated 38.1% mediated despite a near-null NIE, and ALT reached 43.2% under expanded adjustment. The NIE and total effect, with their uncertainty intervals, are therefore more interpretable than the proportion mediated. Expanded adjustment attenuated the total effect on fibrosis by about one-third (from −0.199 to −0.132) but left the NIEs near zero, supporting the primary conclusion without yielding stronger causal estimates, since several of the added covariates may be downstream variables.

These results support conditional rather than universal guidance. Investigators should define the mixture intervention and outcome scale before modeling and use an estimator that targets that contrast directly; any binary-treatment estimator should be evaluated against its own estimand. They should also assess cross-world support in the analytic data (Section 2.5.1 and Figure 2). Where support was sparse and an exposure–mediator interaction plausible, BKMR-CMA was better calibrated in our simulations; where support was adequate, as in NHANES, BART-CMA was more accurate. An application-informed plasmode is most useful for showing how candidate estimators perform under the observed data structure, rather than for selecting the least-biased estimator, because calibrated simulations may not reliably identify it [62]. The total effect should be reported alongside the direct and indirect effects because both estimators could misallocate effects between pathways. Known-truth checks also belong in applied work: an implementation error in BART-CMA, unpaired posterior and mediator draws, produced plausible but overconfident output [25].

Computational cost constrains these choices less than the study’s total runtime might suggest. A single analysis took from approximately 1.5 min to under 4 h; the total of approximately 2220 h arose from repeating each fit across 100 replications, and replications, imputations, and outcomes are independent and can be run in parallel. BKMR-CMA was faster than BART-CMA in 16 of the 17 synthetic scenarios, the exception being a binary outcome at *n* = 1000, where its runtime rose more steeply with sample size. The two methods scale differently. Each BKMR iteration inverts *n* × *n* matrices, a cost that grows with the cube of *n*, which the Gaussian predictive process reduces by replacing them with 100 × 100 matrices [34,36]; a BART iteration grows at least linearly with *n* and is close to independent of the number of predictors [38]. BART-CMA was slower in part because its g-computation averages predictions over all *n* confounder profiles (3000 per replication), whereas BKMR-CMA evaluates counterfactuals at the mean confounder vector [14]. Runtime increases less with mixture size than with sample size, suggesting that larger mixtures are primarily a statistical challenge because the data cover less of the joint exposure space and cross-world predictions rely more on extrapolation than computation. BART-CMA’s burden can be reduced by using fewer retained posterior draws, mediator realizations or trees, at the cost of additional Monte Carlo error or possible loss of accuracy [63].

### 4.1. Limitations

This study has limitations. Because NHANES is cross-sectional, exposures, diet, and liver measures were recorded at a single visit, so the temporal order assumed in Figure 1 cannot be established. This limits interpretation of the sequence mixture → DII → liver outcome in these participants. Diet may contribute to PFAS and metal body burden as readily as it responds to it, and kidney function and liver disease may alter how these chemicals are eliminated, so reverse causation, residual confounding, and selection may contribute to the estimated associations, including the inverse total effect on liver fibrosis. DII was derived from at most two 24 h dietary recalls, and liver enzymes and elastography were measured at a single time point, so biomarker-based exposure assessment may not fully capture relevant chronic exposure windows or etiologically important timing. Causal mediation also requires no unmeasured exposure–outcome, exposure–mediator, or mediator–outcome confounding and no exposure-induced mediator–outcome confounder [25,26,31,64]. These assumptions cannot be verified from the observed data. The synthetic design was not fully factorial because of computational constraints, so we do not interpret the binary-outcome and mixture-size scenarios as marginal main effects across all possible data-generating mechanisms. The fixed-signal scenarios examined only sparse mixtures in which the added components were inert. BKMR-CMA variable selection was not computationally stable in the binary probit analyses and was disabled in that arm, although a sensitivity analysis with selection also disabled in the continuous arm showed that this did not account for the difference between outcome types (Section 3.1.6). Findings may also depend on priors, tree specifications, kernel approximation, MCMC settings, and other implementation choices. Finally, plasmode preserves the observed exposure and confounder dependence but cannot reproduce the unknown causal structure of the NHANES population, because the mediator and outcome models are imposed rather than observed. With 100 replications per scenario, the Monte Carlo standard error of coverage was about 2.2 percentage points, so small differences between methods are hard to resolve, although the differences on which our conclusions rest were at least 3.7 times their Monte Carlo standard errors. Finally, the application used one NHANES cycle, one five-component mixture, and one continuous mediator in US adults, and estimator performance may differ for other mixtures, populations, binary or multiple mediators, and time-to-event outcomes. These constraints motivate the longitudinal and methodological extensions described in Section 4.2.

### 4.2. Future Directions

For future environmental-mixture mediation studies, investigators should define the mixture intervention and outcome scale before modeling, use methods that directly target the resulting estimand, and report support diagnostics and implementation details. BKMR-CMA may be preferable for nonlinear or interaction-rich mixtures in which several components carry mediated effects, or when cross-world support is sparse, if computational resources are sufficient. BART-CMA may suit continuous outcomes and settings with adequate support, particularly when plasmode analyses show strong performance under the intended data structure. Longitudinal studies with repeated measures of exposure, diet, inflammatory status, and liver outcomes would better clarify temporality and determine whether diet-related inflammation mediates longer-term hepatotoxic effects of environmental mixtures. Also, the estimand-aligned comparison could be extended to other classes of mediation estimators once they are adapted to a prespecified intervention on a multivariate continuous exposure.

## 5. Conclusions

We compared BKMR-CMA, BART-CMA, and DeepMed for mediation analysis of environmental mixtures. BKMR-CMA and BART-CMA were evaluated for a prespecified continuous joint-mixture intervention, whereas DeepMed required a binary exposure definition and therefore targeted a different estimand; its indirect-effect estimates were severely biased against both the target truth and the truth for its own binary contrast, so the error reflected estimation failure in this configuration more than the change of estimand. BART-CMA performed well in less complex settings but showed greater bias and undercoverage when exposure–mediator interaction, sparse cross-world support, or additional mixture components carrying effects were present, whereas BKMR-CMA more reliably accommodated nonlinear and interactive mixture-response surfaces, at lower computational cost in all but one scenario. In the NHANES-based plasmode, which matches the application’s support geometry, BART-CMA recovered both the null and the non-null indirect effect with less bias and narrower intervals than BKMR-CMA, although neither method could distinguish an indirect effect of −0.10 outcome standard deviations from zero in samples of 1000. In the applied NHANES analysis, there was little evidence that dietary inflammatory potential mediated associations between the joint PFAS–metal mixture and liver outcomes, although a small indirect effect cannot be ruled out. These findings underscore the importance of defining the mixture intervention and causal estimand before analysis, evaluating both bias and uncertainty calibration, assessing cross-world support, and using application-informed simulations to guide method selection.

## Supplementary Material

Supplementary

**Supplementary Materials:** The following supporting information can be downloaded at https://www.mdpi.com/article/10.3390/stats9060121/s1 , Table S1: Performance for the total, natural direct, and natural indirect effects across the synthetic scenarios; Table S2: DeepMed performance under the quartile binarization; Table S3: BKMR-CMA with componentwise variable selection disabled in scenarios 1, 7, and 8; Table S4: Decomposition of the DeepMed natural indirect effect bias into estimand mismatch and estimation error; Table S5: Cross-world support diagnostics for every setting; Figure S1: DeepMed bias in the natural indirect effect; Figure S2: DeepMed relative bias in the natural indirect effect; Figure S3: DeepMed coverage of the 95% interval; Figure S4: DeepMed interval width; Figure S5: DeepMed estimated indirect effect against the truth; Figure S6: DeepMed bias in all three estimands; Figure S7: Spearman correlation matrix including the primary confounder covariates.

## Figures and Tables

**Figure 1.**
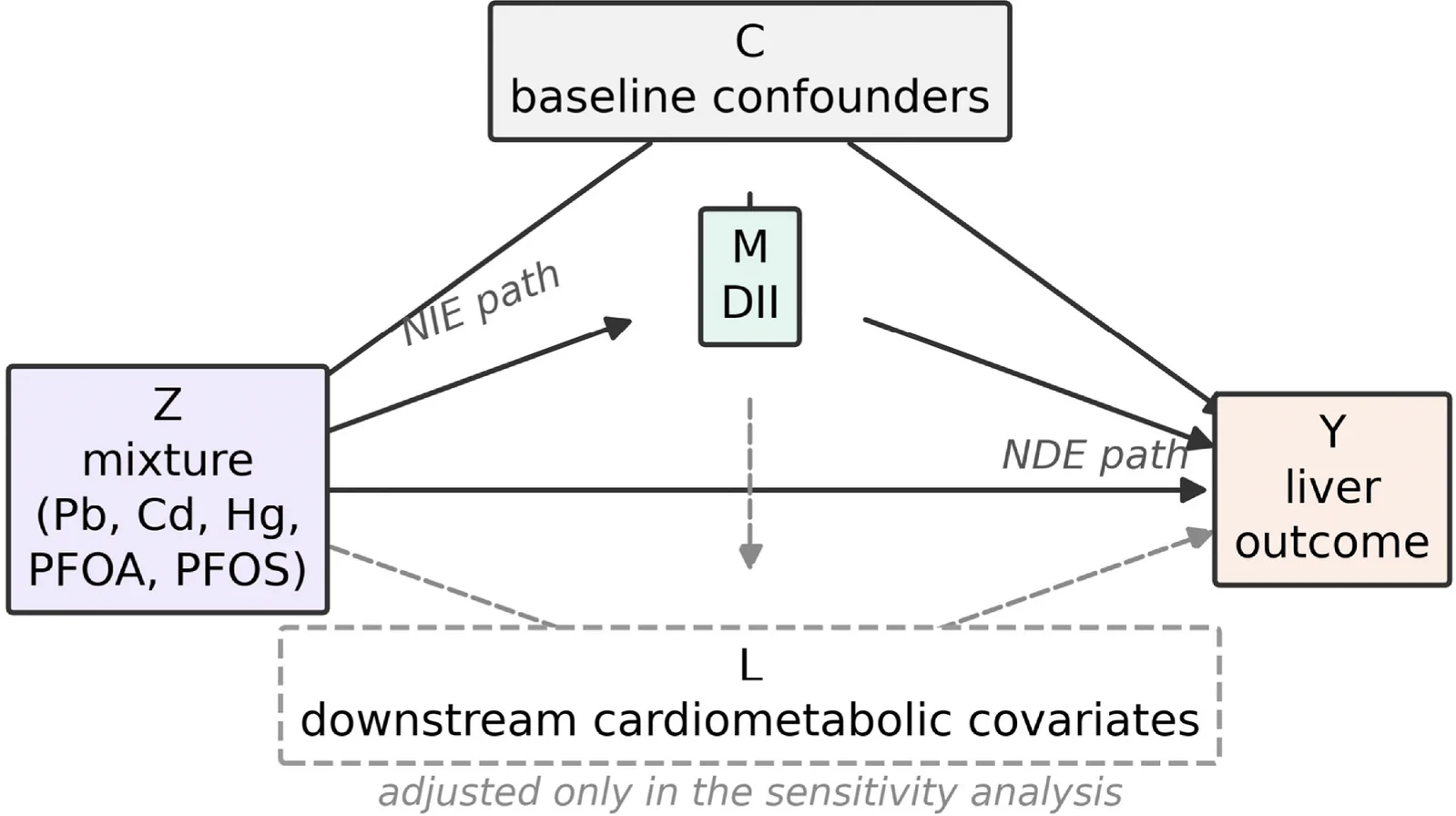
Assumed causal structure for the mixture mediation analysis. Z is the five-component exposure mixture, M is the Dietary Inflammatory Index, Y is a liver outcome, and C is the baseline confounder set. The natural indirect effect is transmitted along Z → M → Y and the natural direct effect along Z → Y. The dashed node L collects the downstream cardiometabolic covariates (waist circumference, C-reactive protein, HbA1c, lipids and blood pressure), which are plausibly affected by both Z and M; adjusting for them blocks part of the mediated pathway, so they enter only the sensitivity analysis of Section 2.10.7. Solid arrows denote the paths represented in the primary analysis and grey dashed arrows the paths involving L, which enter only the sensitivity analysis. The diagram states the assumed ordering; the cross-sectional data (Section 4) do not establish it.

**Figure 2.**
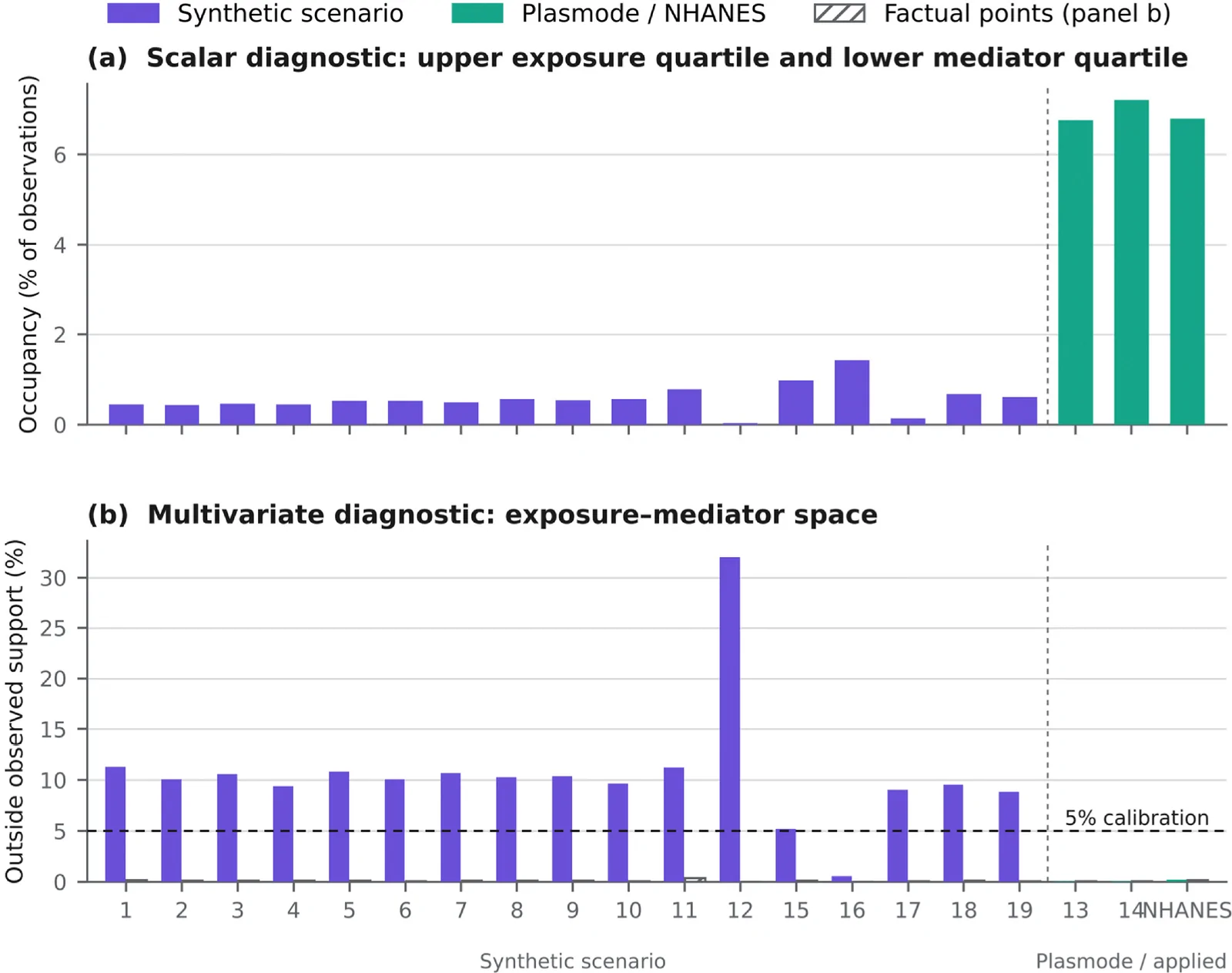
Cross-world support across the seventeen synthetic scenarios, the two plasmode cells, and the NHANES analytic sample. (**a**) Scalar diagnostic: the percentage of observations lying jointly in the upper exposure quartile of the mixture index and the lower mediator quartile. (**b**) Multivariate diagnostic: the percentage of cross-world points ($z_{1},{\;m}_{0}$) classified as outside the observed support in the whitened (L + 1)-dimensional exposure–mediator space, with the factual points shown for comparison. The dashed line marks the 5% of points that the rule classifies as outside by construction. Purple bars denote the synthetic scenarios and green bars the plasmode cells and the NHANES analytic sample; the hatched bars in (**b**) are the factual points. Numerical values are given in Supplementary Table S5.

**Figure 3.**
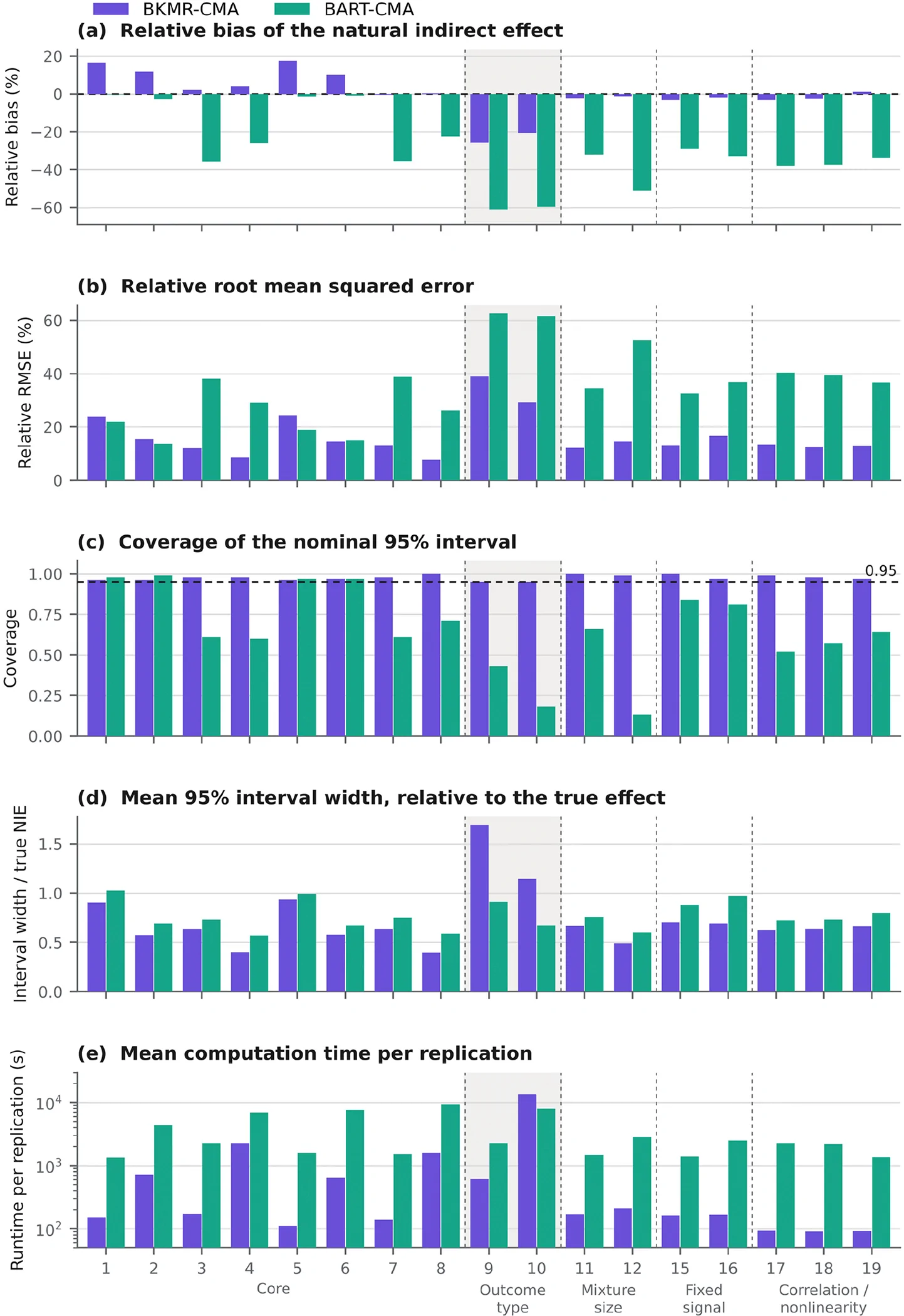
Performance for the natural indirect effect across the seventeen synthetic scenarios. (**a**) Relative bias, the bias as a percentage of the true effect; (**b**) relative root mean squared error; (**c**) coverage of the nominal 95% interval, with the dashed line at the 0.95 target; (**d**) mean 95% interval width divided by the true effect; (**e**) mean computation time per replication, on a logarithmic scale. Bias, RMSE, and width are expressed relative to the true effect because the true natural indirect effect differs across scenarios, ranging from 0.195 to 4.633; absolute values are given in Table 2. The shaded cells (scenarios 9 and 10) have a binary outcome, so their effects are risk differences rather than differences on the continuous outcome scale. Dashed vertical rules separate the design arms, which are named beneath the horizontal axis.

**Figure 4.**
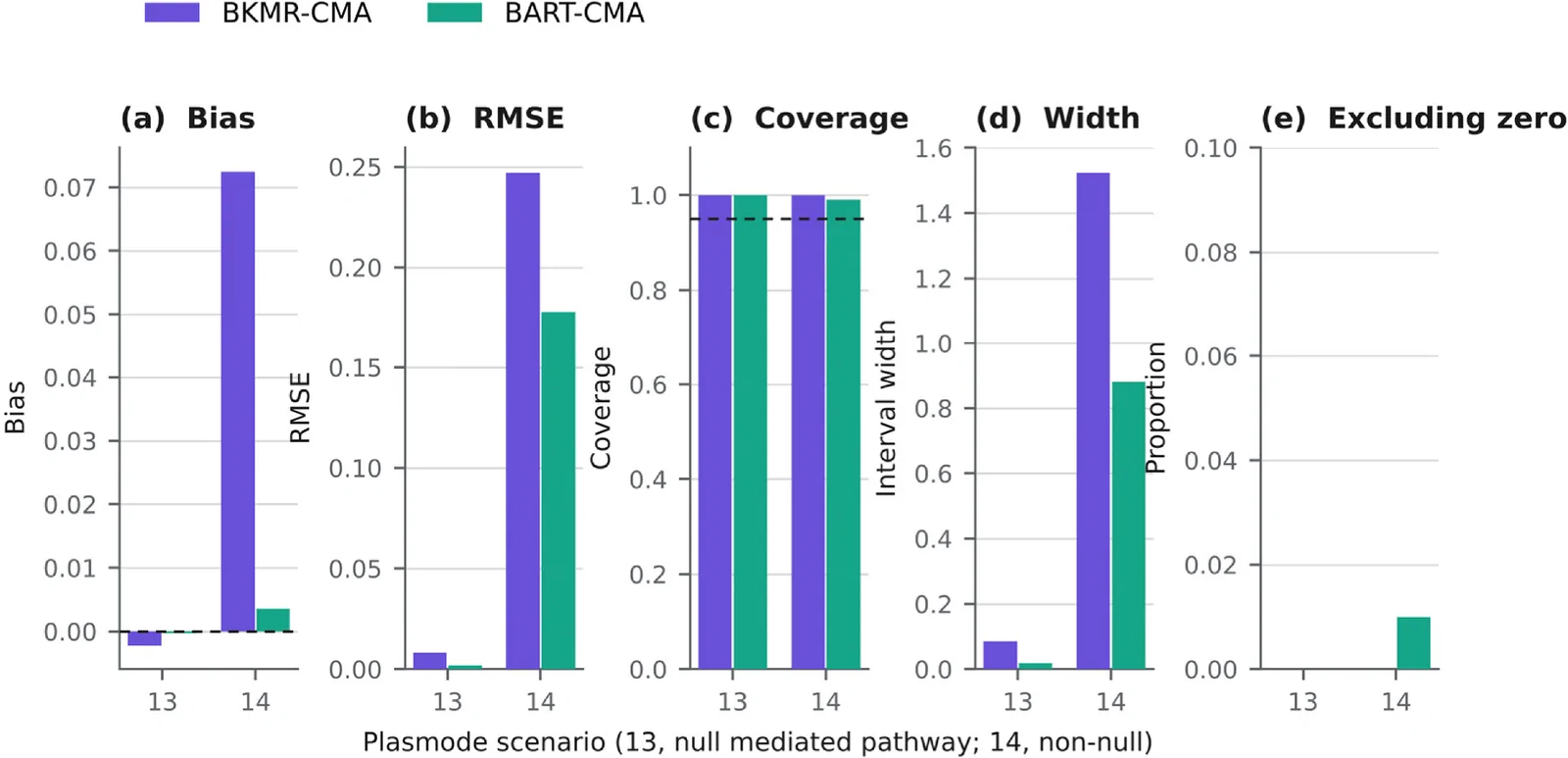
Performance for the natural indirect effect in the two NHANES-based plasmode cells. (a) Bias; (b) root mean squared error; (c) coverage of the nominal 95% interval, with the dashed line at the 0.95 target; (d) mean 95% interval width; (e) proportion of replications whose 95% interval excluded zero. Scenario 13 imposes an effectively null mediated pathway, and scenario 14 a non-null pathway with a true NIE of −0.0394 on the outcome scale. All quantities are on the outcome scale and are also given in Table 3.

**Figure 5.**
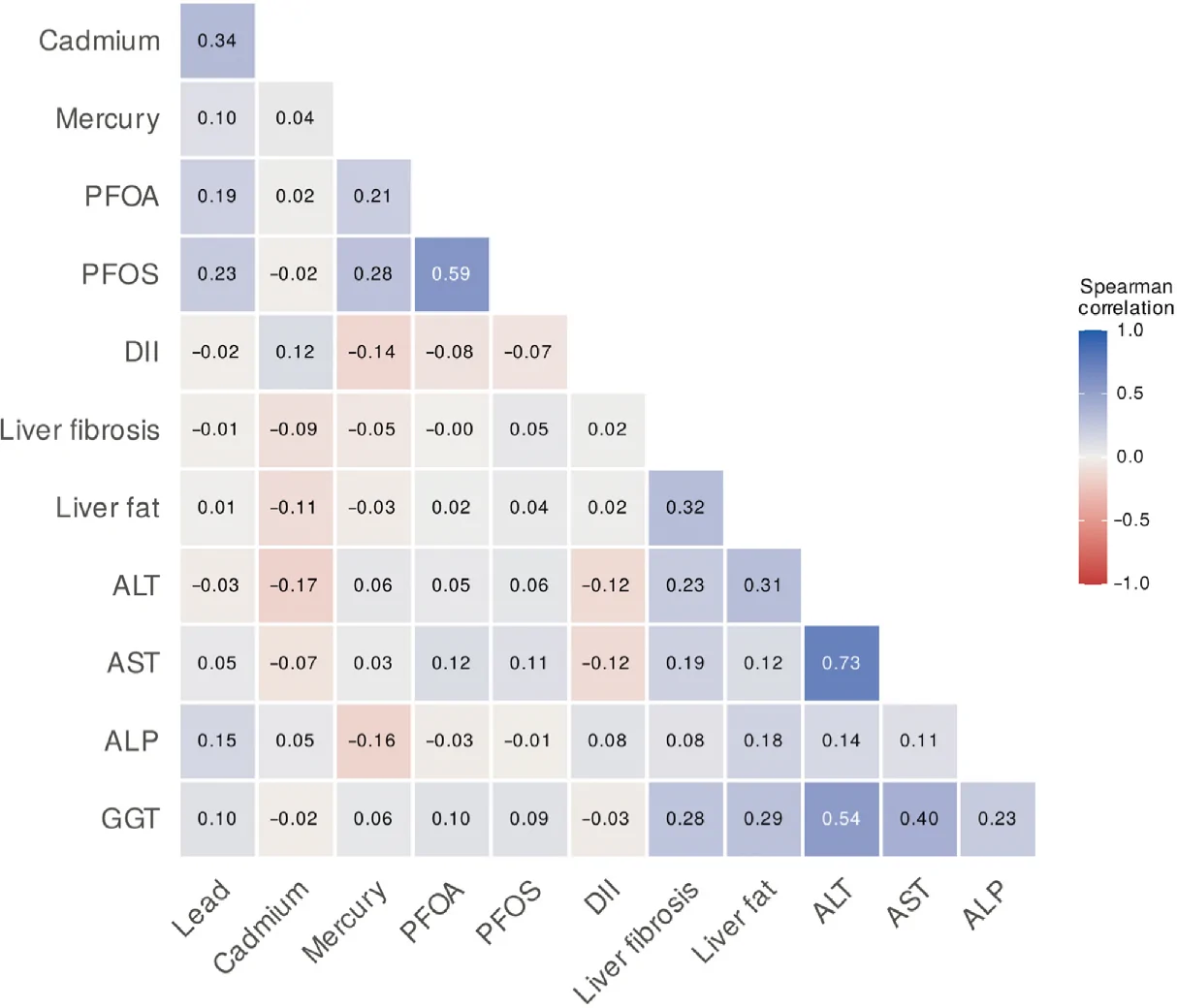
Spearman correlation matrix for the log-transformed exposure biomarkers (lead, cadmium, mercury, PFOA, PFOS), the Dietary Inflammatory Index (DII), and the six liver outcomes (liver fibrosis, liver fat, ALT, AST, alkaline phosphatase, GGT) among the 1498 adults in the NHANES 2017–2018 PFAS subsample. Correlations are pooled across the 20 imputed datasets.

**Figure 6.**
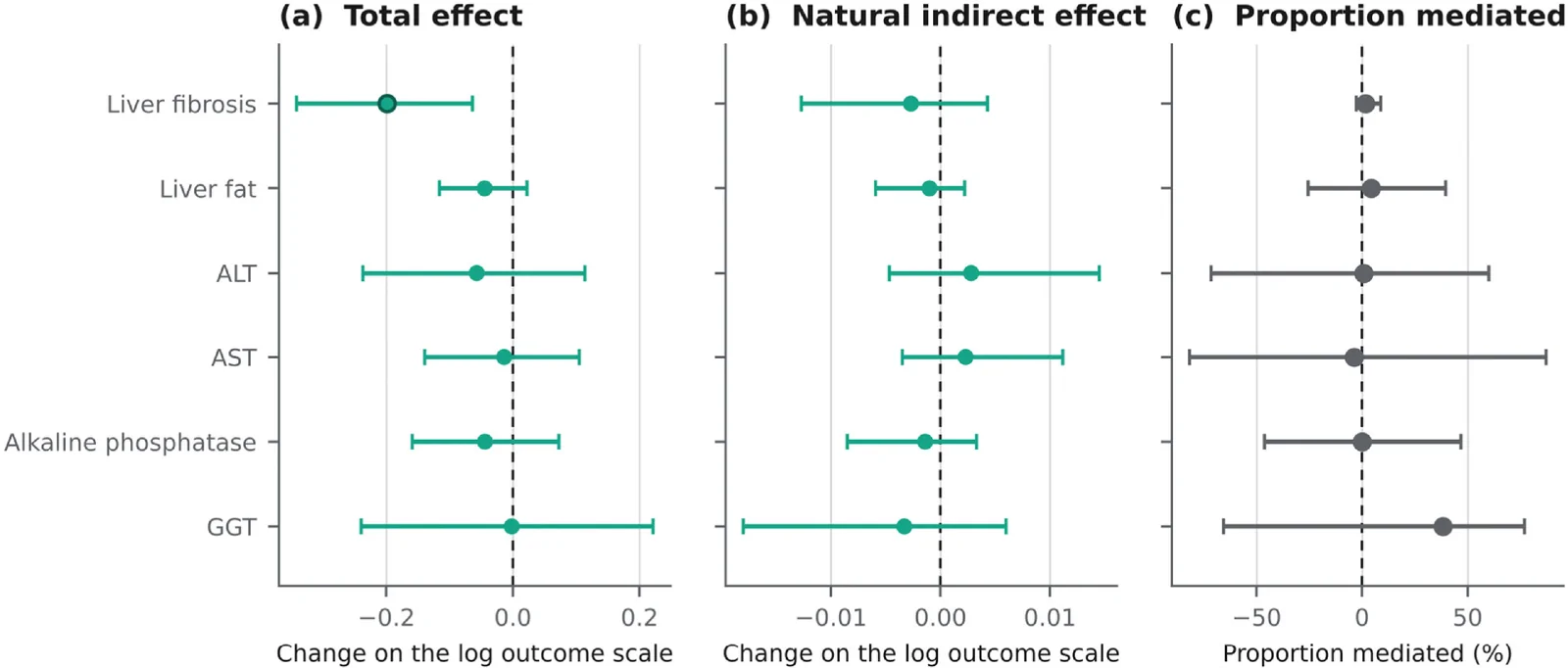
Applied BART-CMA estimates for a simultaneous shift of every component of the PFAS–metal mixture from its 25th to its 75th percentile, primary confounder set, among 1498 adults. (**a**) Total effect; (**b**) natural indirect effect through the Dietary Inflammatory Index; (**c**) proportion mediated. Points are pooled posterior means across the 20 imputed datasets and bars are 95% credible intervals; outcomes in (**a**,**b**) are on the log scale. A darker marker outline marks an interval excluding zero. The ratio NIE/TE in (**c**) is heavy-tailed when the total effect is near zero, so the posterior mean need not match the ratio of the NIE and TE point estimates. Numerical values are given in Table 4.

**Figure 7.**
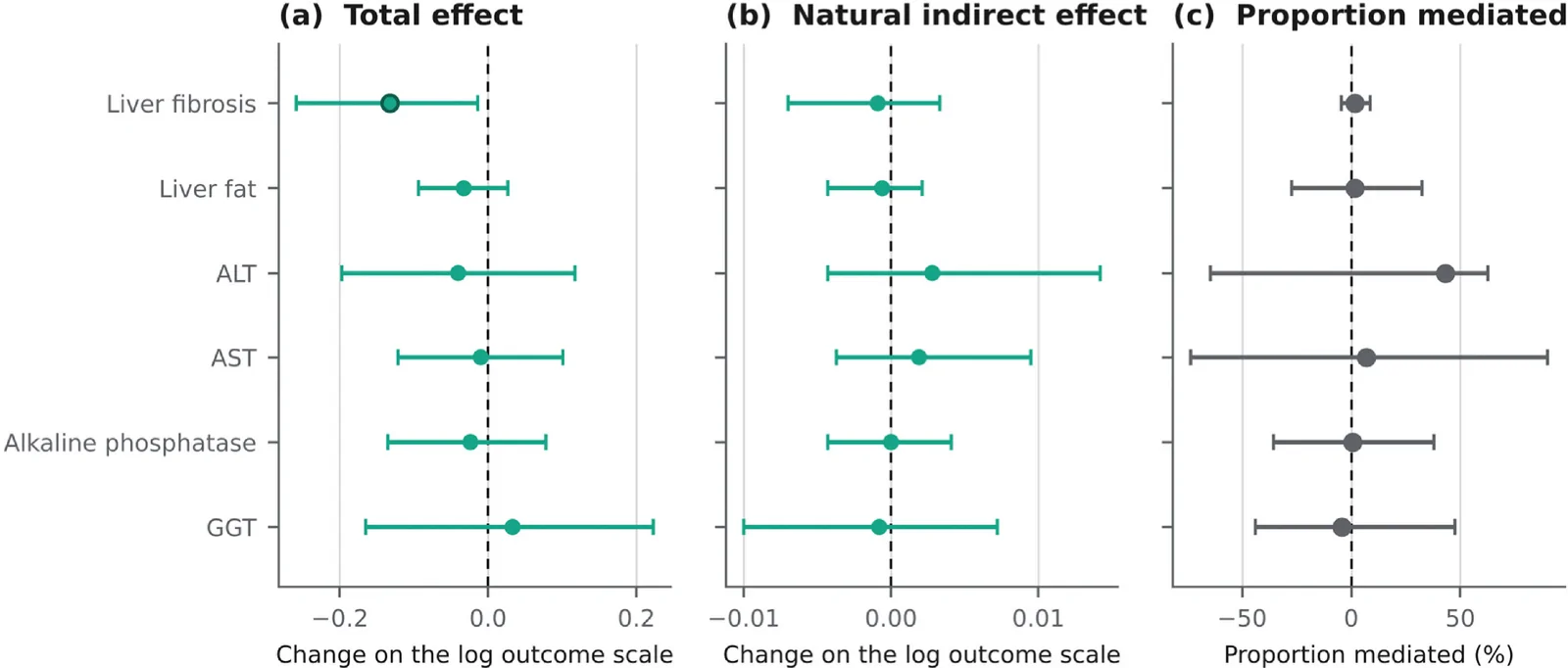
Applied BART-CMA estimates under the expanded confounder set (over-adjustment sensitivity analysis). (**a**) Total effect; (**b**) natural indirect effect through the Dietary Inflammatory Index; (**c**) proportion mediated. Panels, markers and intervals are as in Figure 6. Numerical values are given in Table 5.

**Table 1.** Simulation design. Scenarios 1–8 form a fully crossed core; scenarios 9–12 and 15–19 are targeted extensions anchored to the nonlinear, interaction-present condition.

Scenario	Linearity	Z × M Interaction	Outcome	*n*	L	Active Exposures	Arm
1	Linear	None	Continuous	300	5	5	Core
2	Linear	None	Continuous	1000	5	5	Core
3	Linear	Present	Continuous	300	5	5	Core
4	Linear	Present	Continuous	1000	5	5	Core
5	Nonlinear	None	Continuous	300	5	5	Core
6	Nonlinear	None	Continuous	1000	5	5	Core
7	Nonlinear	Present	Continuous	300	5	5	Core
8	Nonlinear	Present	Continuous	1000	5	5	Core
9	Nonlinear	Present	Binary	300	5	5	Outcome type
10	Nonlinear	Present	Binary	1000	5	5	Outcome type
11	Nonlinear	Present	Continuous	300	3	3	Mixture size
12	Nonlinear	Present	Continuous	300	10	10	Mixture size
15	Nonlinear	Present	Continuous	300	5	3	Mixture size (fixed signal)
16	Nonlinear	Present	Continuous	300	10	3	Mixture size (fixed signal)
17	Nonlinear	Present	Continuous	300	5	5	Exposure correlation (0.7)
18	Nonlinear	Present	Continuous	300	5	5	Exposure correlation (block 0.7/0.3/0.1)
19	Nonlinear	Present	Continuous	300	5	5	Stronger nonlinearity (×2)

Active exposures: number of exposures with nonzero mediator, outcome, and interaction coefficients. In Scenarios 15–16, the remaining L − 3 exposures are generated identically to the others but have zero coefficients (Section 2.5.2). Scenarios 13–14 form the plasmode arm (Section 2.5.1).

**Table 2.** Performance for the natural indirect effect across seventeen synthetic scenarios. Bias, RMSE, and width are on the outcome scale; MCSE is the Monte Carlo standard error of the bias; runtime is the mean per replication. Fits is the number of replications out of 100 that produced a usable estimate, and all performance measures are computed over those replications.

Scenario	Method	Fits	Truth	Bias	MCSE	RMSE	Coverage	Width	Runtime (s)
1	BKMR-CMA	100	1.102	0.182	0.019	0.263	0.96	0.998	152
	BART-CMA	100	1.102	−0.002	0.024	0.242	0.98	1.131	1329
2	BKMR-CMA	100	1.102	0.131	0.011	0.169	0.96	0.631	715
	BART-CMA	100	1.102	−0.031	0.015	0.151	0.99	0.761	4395
3	BKMR-CMA	100	1.862	0.041	0.022	0.225	0.98	1.181	171
	BART-CMA	100	1.862	−0.665	0.025	0.709	0.61	1.361	2262
4	BKMR-CMA	100	1.862	0.077	0.014	0.159	0.98	0.742	2271
	BART-CMA	100	1.862	−0.483	0.025	0.542	0.60	1.059	6881
5	BKMR-CMA	100	1.158	0.203	0.019	0.280	0.96	1.083	111
	BART-CMA	100	1.158	−0.017	0.022	0.219	0.97	1.149	1575
6	BKMR-CMA	100	1.158	0.119	0.012	0.168	0.97	0.671	635
	BART-CMA	100	1.158	−0.010	0.017	0.174	0.97	0.777	7596
7	BKMR-CMA	100	1.958	−0.009	0.026	0.259	0.98	1.240	137
	BART-CMA	100	1.958	−0.696	0.031	0.760	0.61	1.475	1520
8	BKMR-CMA	100	1.958	0.006	0.015	0.150	1.00	0.776	1586
	BART-CMA	100	1.958	−0.436	0.027	0.514	0.71	1.151	9277
9	BKMR-CMA	100	0.195	−0.050	0.006	0.076	0.95	0.330	613
	BART-CMA	100	0.195	−0.119	0.003	0.122	0.43	0.178	2248
10	BKMR-CMA	97	0.195	−0.040	0.004	0.057	0.95	0.223	13,370
	BART-CMA	100	0.195	−0.116	0.003	0.120	0.18	0.131	7930
11	BKMR-CMA	100	1.546	−0.038	0.019	0.189	1.00	1.035	168
	BART-CMA	100	1.546	−0.495	0.020	0.533	0.66	1.175	1479
12	BKMR-CMA	100	4.633	−0.058	0.067	0.674	0.99	2.269	209
	BART-CMA	100	4.633	−2.369	0.058	2.438	0.13	2.789	2827
15	BKMR-CMA	100	1.544	−0.048	0.020	0.203	1.00	1.088	161
	BART-CMA	100	1.544	−0.448	0.023	0.505	0.84	1.358	1408
16	BKMR-CMA	100	1.547	−0.030	0.026	0.258	0.97	1.068	166
	BART-CMA	100	1.547	−0.508	0.026	0.570	0.81	1.503	2480
17	BKMR-CMA	100	1.961	−0.062	0.026	0.263	0.99	1.219	92
	BART-CMA	100	1.961	−0.746	0.027	0.792	0.52	1.418	2257
18	BKMR-CMA	100	1.960	−0.052	0.024	0.245	0.98	1.248	90
	BART-CMA	100	1.960	−0.733	0.024	0.772	0.57	1.428	2209
19	BKMR-CMA	100	1.958	0.021	0.025	0.251	0.97	1.300	91
	BART-CMA	100	1.958	−0.658	0.029	0.719	0.64	1.562	1350

**Table 3.** Performance in the two plasmode cells. Scenario 13 imposes an effectively null mediated pathway and scenario 14 a non-null; exposures and confounders are resampled from the NHANES analytic dataset. The true NIE in scenario 14 corresponds to the imposed effect of −0.10 outcome standard deviations. Columns are defined as in Table 2.

Scenario	Estimand	Method	Fits	Truth	Bias	MCSE	RMSE	Coverage	Width	Excl. 0
13 (null)	TE	BKMR-CMA	100	−0.0719	0.0501	0.0023	0.0552	0.38	0.0739	0.09
		BART-CMA	100	−0.0719	−0.0020	0.0060	0.0600	0.98	0.3064	0.08
	NDE	BKMR-CMA	100	−0.0719	0.0524	0.0023	0.0572	0.32	0.0878	0.13
		BART-CMA	100	−0.0719	−0.0017	0.0060	0.0598	0.98	0.3065	0.10
	NIE	BKMR-CMA	100	−0.0000	−0.0022	0.0008	0.0083	1.00	0.0848	0.00
		BART-CMA	100	−0.0000	−0.0003	0.0002	0.0017	1.00	0.0176	0.00
14 (non-null)	TE	BKMR-CMA	100	−0.3327	−0.0268	0.0462	0.4602	0.97	2.1881	0.05
		BART-CMA	100	−0.3327	−0.1549	0.0844	0.8541	0.99	3.7321	0.08
	NDE	BKMR-CMA	100	−0.2932	−0.0992	0.0366	0.3772	0.92	1.3307	0.22
		BART-CMA	100	−0.2932	−0.1585	0.0721	0.7348	0.97	3.1067	0.09
	NIE	BKMR-CMA	100	−0.0394	0.0725	0.0237	0.2471	1.00	1.5243	0.00
		BART-CMA	100	−0.0394	0.0036	0.0179	0.1777	0.99	0.8794	0.01

**Table 4.** Mediation estimates for the PFAS–metal mixture and the six liver outcomes under the primary confounder set. Estimates are pooled posterior means with 95% credible intervals for a simultaneous shift of every mixture component from its 25th to its 75th percentile; outcomes are on the log scale. SD of PM is the posterior standard deviation of the proportion mediated, on the proportion scale, reported because the ratio NIE/TE is heavy-tailed when the total effect is near zero.

Outcome	TE (95% CrI)	NDE (95% CrI)	NIE (95% CrI)	PM, % (95% CrI)	SD of PM
Liver fibrosis	−0.1986 (−0.3416, −0.0642)	−0.1959 (−0.3408, −0.0613)	−0.0027 (−0.0127, 0.0043)	1.7 (−2.6, 9.0)	0.05
Liver fat	−0.0447 (−0.1163, 0.0224)	−0.0437 (−0.1148, 0.0233)	−0.0010 (−0.0059, 0.0022)	4.4 (−25.7, 39.6)	0.70
ALT	−0.0572 (−0.2372, 0.1134)	−0.0599 (−0.2401, 0.1100)	0.0028 (−0.0047, 0.0145)	0.7 (−71.5, 60.1)	1.46
AST	−0.0139 (−0.1388, 0.1049)	−0.0162 (−0.1394, 0.1028)	0.0023 (−0.0035, 0.0112)	−3.7 (−81.7, 87.1)	3.96
Alkaline phosphatase	−0.0442 (−0.1589, 0.0729)	−0.0428 (−0.1584, 0.0739)	−0.0014 (−0.0085, 0.0033)	−0.1 (−46.2, 46.7)	1.10
GGT	−0.0019 (−0.2400, 0.2210)	0.0014 (−0.2356, 0.2245)	−0.0033 (−0.0180, 0.0060)	38.1 (−65.6, 77.1)	22.1

**Table 5.** Mediation estimates under the expanded confounder set (over-adjustment sensitivity analysis), which additionally includes waist circumference, C-reactive protein, HbA1c, total and HDL cholesterol, and systolic and diastolic blood pressure. Columns are defined as in Table 4.

Outcome	TE (95% CrI)	NDE (95% CrI)	NIE (95% CrI)	PM, % (95% CrI)	SD of PM
Liver fibrosis	−0.1320 (−0.2582, −0.0143)	−0.1311 (−0.2555, −0.0137)	−0.0009 (−0.0070, 0.0033)	1.8 (−4.7, 8.8)	0.64
Liver fat	−0.0327 (−0.0935, 0.0264)	−0.0322 (−0.0938, 0.0275)	−0.0006 (−0.0043, 0.0021)	1.8 (−27.3, 32.4)	0.73
ALT	−0.0404 (−0.1973, 0.1171)	−0.0432 (−0.1994, 0.1130)	0.0028 (−0.0043, 0.0142)	43.2 (−64.6, 62.7)	28.5
AST	−0.0098 (−0.1214, 0.1008)	−0.0117 (−0.1231, 0.0968)	0.0019 (−0.0037, 0.0095)	6.8 (−73.5, 89.9)	2.96
Alkaline phosphatase	−0.0237 (−0.1349, 0.0782)	−0.0237 (−0.1341, 0.0771)	0.0000 (−0.0043, 0.0041)	0.6 (−35.7, 37.8)	0.62
GGT	0.0332 (−0.1649, 0.2226)	0.0340 (−0.1638, 0.2227)	−0.0008 (−0.0100, 0.0072)	−4.4 (−43.9, 47.6)	1.93

## Data Availability

The NHANES dataset is publicly available online, accessible at https://wwwn.cdc.gov/nchs/nhanes/continuousnhanes/overview.aspx?BeginYear=2017 (accessed on 22 June 2026). All R code used for the simulation, plasmode, and applied analyses was written in R 4.5.0 and is openly available in the GitHub repository https://github.com/OdeAugustina/Causal-Mediation-Analysis-of-Environmental-Mixtures (accessed on 27 October 2026) and archived at Zenodo, https://doi.org/10.5281/zenodo.22782980 (version v1.0.0). The repository includes the data-generating, truth, method, and aggregation libraries for the seventeen synthetic scenarios and the two plasmode cells, the SLURM array scripts that reproduce the full pipeline, and the seeds required to reproduce every reported figure and table. The two implementation corrections reported in this paper are provided as standalone, independently checkable scripts: fix_causalbkmr_astar.R and verify_causalbkmr_fix.R implement and verify the correction to the cross-world mediator draw described in Section 2.7.1, and patch_pairing.py with smoke_paired_bart_v2.R implement and check the pairing of mediator draws with their posterior draws described in Section 2.7.2.
