# Supplementary material for: Estimand Alignment and Implementation Sensitivity in Causal Mediation Analysis of Environmental Mixtures: Simulation, Plasmode, and Applied Study
Source: Stats (Basel). Author manuscript; Available in PMC 2026 Nov 8. (PMC13742161; doi:10.3390/stats9060121)
Supplement: Supplementary file 1 [file NIHMS2220696-supplement-Supplementary.pdf]

*Supplementary Materials*

# Estimand Alignment and Implementation Sensitivity in Causal Mediation Analysis of Environmental Mixtures: Simulation, Plasmode, and Applied Study

**Augustina Odediran [1,2] and Emmanuel Obeng-Gyasi [1,2,*]**

[1] Department of Built Environment, North Carolina A&T State University, Greensboro, NC 27411, USA
[2] Environmental Health and Disease Laboratory, North Carolina A&T State University, Greensboro, NC 27411, USA
* Correspondence: eobenggyasi@ncat.edu; Tel.: +1-336-285-3132

## Contents

1. Table S1. Performance for the total, natural direct, and natural indirect effects across the synthetic scenarios.
2. Table S2. DeepMed performance under the quartile binarization.
3. Table S3. BKMR-CMA with componentwise variable selection disabled in scenarios 1, 7, and 8.
4. Table S4. Decomposition of the DeepMed natural indirect effect bias into estimand mismatch and estimation error.
5. Table S5. Cross-world support diagnostics for every setting.
6. Figure S1. DeepMed bias in the natural indirect effect.
7. Figure S2. DeepMed relative bias in the natural indirect effect.
8. Figure S3. DeepMed coverage of the 95% interval.
9. Figure S4. DeepMed interval width.
10. Figure S5. DeepMed estimated indirect effect against the truth.
11. Figure S6. DeepMed bias in all three estimands.
12. Figure S7. Spearman correlation matrix including the primary confounder covariates.

## Supplementary Tables

**Table S1. Performance for the total effect (TE), natural direct effect (NDE), and natural indirect effect (NIE) across the seventeen synthetic scenarios. Bias, RMSE, and width are on the outcome scale; MCSE is the Monte Carlo standard error of the bias; runtime is the mean per replication. Fits is the number of replications out of 100 that produced a usable estimate. Table 1 in the main text defines the scenarios.**

| Scenario | Estimand | Method | Fits | Truth | Bias | MCSE | RMSE | Coverage | Width | Runtime (s) |
|---|---|---|---|---|---|---|---|---|---|---|
| 1 | TE | BKMR-CMA | 100 | 2.548 | 0.122 | 0.014 | 0.184 | 0.95 | 0.683 | 152 |
| | | BART-CMA | 100 | 2.548 | 0.100 | 0.039 | 0.398 | 0.98 | 2.211 | 1,329 |
| | NDE | BKMR-CMA | 100 | 1.446 | −0.060 | 0.022 | 0.225 | 0.93 | 0.919 | 152 |
| | | BART-CMA | 100 | 1.446 | 0.102 | 0.035 | 0.365 | 0.99 | 2.076 | 1,329 |
| | NIE | BKMR-CMA | 100 | 1.102 | 0.182 | 0.019 | 0.263 | 0.96 | 0.998 | 152 |
| | | BART-CMA | 100 | 1.102 | −0.002 | 0.024 | 0.242 | 0.98 | 1.131 | 1,329 |
| 2 | TE | BKMR-CMA | 100 | 2.548 | 0.126 | 0.010 | 0.160 | 0.72 | 0.386 | 715 |
| | | BART-CMA | 100 | 2.548 | −0.022 | 0.033 | 0.332 | 0.98 | 1.510 | 4,395 |
| | NDE | BKMR-CMA | 100 | 1.446 | −0.005 | 0.014 | 0.138 | 0.98 | 0.585 | 715 |
| | | BART-CMA | 100 | 1.446 | 0.008 | 0.029 | 0.285 | 0.97 | 1.384 | 4,395 |
| | NIE | BKMR-CMA | 100 | 1.102 | 0.131 | 0.011 | 0.169 | 0.96 | 0.631 | 715 |
| | | BART-CMA | 100 | 1.102 | −0.031 | 0.015 | 0.151 | 0.99 | 0.761 | 4,395 |
| 3 | TE | BKMR-CMA | 100 | 2.897 | 0.131 | 0.019 | 0.230 | 0.90 | 0.786 | 171 |
| | | BART-CMA | 100 | 2.897 | 0.024 | 0.047 | 0.464 | 0.99 | 2.578 | 2,262 |

| Scenario | Estimand | Method | Fits | Truth | Bias | MCSE | RMSE | Coverage | Width | Runtime (s) |
|---|---|---|---|---|---|---|---|---|---|---|
| | NDE | BKMR-CMA | 100 | 1.035 | 0.090 | 0.022 | 0.238 | 0.99 | 1.065 | 171 |
| | | BART-CMA | 100 | 1.035 | 0.689 | 0.044 | 0.818 | 0.87 | 2.441 | 2,262 |
| | NIE | BKMR-CMA | 100 | 1.862 | 0.041 | 0.022 | 0.225 | 0.98 | 1.181 | 171 |
| | | BART-CMA | 100 | 1.862 | −0.665 | 0.025 | 0.709 | 0.61 | 1.361 | 2,262 |
| 4 | TE | BKMR-CMA | 100 | 2.897 | 0.090 | 0.012 | 0.147 | 0.87 | 0.439 | 2,271 |
| | | BART-CMA | 100 | 2.897 | 0.001 | 0.034 | 0.341 | 0.99 | 1.666 | 6,881 |
| | NDE | BKMR-CMA | 100 | 1.035 | 0.013 | 0.014 | 0.140 | 0.99 | 0.691 | 2,271 |
| | | BART-CMA | 100 | 1.035 | 0.484 | 0.034 | 0.591 | 0.84 | 1.584 | 6,881 |
| | NIE | BKMR-CMA | 100 | 1.862 | 0.077 | 0.014 | 0.159 | 0.98 | 0.742 | 2,271 |
| | | BART-CMA | 100 | 1.862 | −0.483 | 0.025 | 0.542 | 0.60 | 1.059 | 6,881 |
| 5 | TE | BKMR-CMA | 100 | 2.690 | −0.046 | 0.019 | 0.195 | 0.95 | 0.752 | 111 |
| | | BART-CMA | 100 | 2.690 | −0.011 | 0.046 | 0.461 | 0.99 | 2.268 | 1,575 |
| | NDE | BKMR-CMA | 100 | 1.532 | −0.249 | 0.025 | 0.355 | 0.80 | 0.985 | 111 |
| | | BART-CMA | 100 | 1.532 | 0.006 | 0.045 | 0.447 | 0.99 | 2.134 | 1,575 |
| | NIE | BKMR-CMA | 100 | 1.158 | 0.203 | 0.019 | 0.280 | 0.96 | 1.083 | 111 |
| | | BART-CMA | 100 | 1.158 | −0.017 | 0.022 | 0.219 | 0.97 | 1.149 | 1,575 |
| 6 | TE | BKMR-CMA | 100 | 2.690 | −0.069 | 0.010 | 0.124 | 0.92 | 0.439 | 635 |
| | | BART-CMA | 100 | 2.690 | 0.017 | 0.033 | 0.328 | 0.98 | 1.499 | 7,596 |
| | NDE | BKMR-CMA | 100 | 1.532 | −0.187 | 0.012 | 0.223 | 0.86 | 0.611 | 635 |
| | | BART-CMA | 100 | 1.532 | 0.028 | 0.027 | 0.266 | 0.98 | 1.395 | 7,596 |
| | NIE | BKMR-CMA | 100 | 1.158 | 0.119 | 0.012 | 0.168 | 0.97 | 0.671 | 635 |
| | | BART-CMA | 100 | 1.158 | −0.010 | 0.017 | 0.174 | 0.97 | 0.777 | 7,596 |
| 7 | TE | BKMR-CMA | 100 | 3.088 | −0.066 | 0.023 | 0.236 | 0.94 | 0.873 | 137 |
| | | BART-CMA | 100 | 3.088 | 0.025 | 0.046 | 0.463 | 0.98 | 2.727 | 1,520 |
| | NDE | BKMR-CMA | 100 | 1.131 | −0.057 | 0.025 | 0.259 | 0.93 | 1.111 | 137 |
| | | BART-CMA | 100 | 1.131 | 0.721 | 0.045 | 0.847 | 0.92 | 2.570 | 1,520 |
| | NIE | BKMR-CMA | 100 | 1.958 | −0.009 | 0.026 | 0.259 | 0.98 | 1.240 | 137 |
| | | BART-CMA | 100 | 1.958 | −0.696 | 0.031 | 0.760 | 0.61 | 1.475 | 1,520 |
| 8 | TE | BKMR-CMA | 100 | 3.088 | −0.108 | 0.013 | 0.170 | 0.83 | 0.509 | 1,586 |
| | | BART-CMA | 100 | 3.088 | −0.017 | 0.038 | 0.377 | 0.97 | 1.697 | 9,277 |
| | NDE | BKMR-CMA | 100 | 1.131 | −0.113 | 0.017 | 0.201 | 0.94 | 0.706 | 1,586 |
| | | BART-CMA | 100 | 1.131 | 0.419 | 0.038 | 0.566 | 0.83 | 1.636 | 9,277 |
| | NIE | BKMR-CMA | 100 | 1.958 | 0.006 | 0.015 | 0.150 | 1.00 | 0.776 | 1,586 |
| | | BART-CMA | 100 | 1.958 | −0.436 | 0.027 | 0.514 | 0.71 | 1.151 | 9,277 |
| 9 | TE | BKMR-CMA | 100 | 0.395 | −0.051 | 0.006 | 0.078 | 0.91 | 0.281 | 613 |
| | | BART-CMA | 100 | 0.395 | −0.036 | 0.007 | 0.082 | 1.00 | 0.460 | 2,248 |
| | NDE | BKMR-CMA | 100 | 0.199 | −0.000 | 0.009 | 0.089 | 1.00 | 0.401 | 613 |
| | | BART-CMA | 100 | 0.199 | 0.082 | 0.008 | 0.118 | 0.98 | 0.529 | 2,248 |
| | NIE | BKMR-CMA | 100 | 0.195 | −0.050 | 0.006 | 0.076 | 0.95 | 0.330 | 613 |
| | | BART-CMA | 100 | 0.195 | −0.119 | 0.003 | 0.122 | 0.43 | 0.178 | 2,248 |
| 10 | TE | BKMR-CMA | 97 | 0.395 | −0.016 | 0.004 | 0.038 | 0.96 | 0.159 | 13,370 |
| | | BART-CMA | 100 | 0.395 | 0.014 | 0.006 | 0.065 | 1.00 | 0.337 | 7,930 |
| | NDE | BKMR-CMA | 97 | 0.199 | 0.024 | 0.005 | 0.057 | 0.98 | 0.262 | 13,370 |
| | | BART-CMA | 100 | 0.199 | 0.130 | 0.007 | 0.148 | 0.84 | 0.391 | 7,930 |
| | NIE | BKMR-CMA | 97 | 0.195 | −0.040 | 0.004 | 0.057 | 0.95 | 0.223 | 13,370 |
| | | BART-CMA | 100 | 0.195 | −0.116 | 0.003 | 0.120 | 0.18 | 0.131 | 7,930 |
| 11 | TE | BKMR-CMA | 100 | 2.640 | −0.073 | 0.020 | 0.212 | 0.92 | 0.776 | 168 |
| | | BART-CMA | 100 | 2.640 | −0.027 | 0.041 | 0.405 | 0.99 | 2.163 | 1,479 |
| | NDE | BKMR-CMA | 100 | 1.094 | −0.035 | 0.021 | 0.216 | 0.95 | 0.920 | 168 |
| | | BART-CMA | 100 | 1.094 | 0.467 | 0.039 | 0.608 | 0.90 | 2.089 | 1,479 |
| | NIE | BKMR-CMA | 100 | 1.546 | −0.038 | 0.019 | 0.189 | 1.00 | 1.035 | 168 |
| | | BART-CMA | 100 | 1.546 | −0.495 | 0.020 | 0.533 | 0.66 | 1.175 | 1,479 |
| 12 | TE | BKMR-CMA | 100 | 5.473 | 0.170 | 0.063 | 0.650 | 0.89 | 1.121 | 209 |
| | | BART-CMA | 100 | 5.473 | −0.000 | 0.084 | 0.832 | 0.96 | 4.274 | 2,827 |
| | NDE | BKMR-CMA | 100 | 0.840 | 0.228 | 0.043 | 0.480 | 1.00 | 2.158 | 209 |
| | | BART-CMA | 100 | 0.840 | 2.369 | 0.077 | 2.488 | 0.26 | 3.876 | 2,827 |
| | NIE | BKMR-CMA | 100 | 4.633 | −0.058 | 0.067 | 0.674 | 0.99 | 2.269 | 209 |
| | | BART-CMA | 100 | 4.633 | −2.369 | 0.058 | 2.438 | 0.13 | 2.789 | 2,827 |
| 15 | TE | BKMR-CMA | 100 | 2.637 | −0.051 | 0.019 | 0.198 | 0.98 | 0.804 | 161 |
| | | BART-CMA | 100 | 2.637 | 0.098 | 0.051 | 0.516 | 0.98 | 2.542 | 1,408 |
| | NDE | BKMR-CMA | 100 | 1.093 | −0.003 | 0.023 | 0.231 | 0.95 | 0.968 | 161 |

| Scenario | Estimand | Method | Fits | Truth | Bias | MCSE | RMSE | Coverage | Width | Runtime (s) |
|---|---|---|---|---|---|---|---|---|---|---|
| | | BART-CMA | 100 | 1.093 | 0.546 | 0.049 | 0.735 | 0.89 | 2.437 | 1,408 |
| | NIE | BKMR-CMA | 100 | 1.544 | −0.048 | 0.020 | 0.203 | 1.00 | 1.088 | 161 |
| | | BART-CMA | 100 | 1.544 | −0.448 | 0.023 | 0.505 | 0.84 | 1.358 | 1,408 |
| 16 | TE | BKMR-CMA | 100 | 2.627 | −0.066 | 0.027 | 0.277 | 0.95 | 0.793 | 166 |
| | | BART-CMA | 100 | 2.627 | −0.053 | 0.058 | 0.577 | 1.00 | 3.088 | 2,480 |
| | NDE | BKMR-CMA | 100 | 1.081 | −0.036 | 0.027 | 0.271 | 0.89 | 0.953 | 166 |
| | | BART-CMA | 100 | 1.081 | 0.455 | 0.054 | 0.704 | 0.97 | 2.912 | 2,480 |
| | NIE | BKMR-CMA | 100 | 1.547 | −0.030 | 0.026 | 0.258 | 0.97 | 1.068 | 166 |
| | | BART-CMA | 100 | 1.547 | −0.508 | 0.026 | 0.570 | 0.81 | 1.503 | 2,480 |
| 17 | TE | BKMR-CMA | 100 | 3.093 | −0.107 | 0.019 | 0.214 | 0.90 | 0.754 | 92 |
| | | BART-CMA | 100 | 3.093 | −0.038 | 0.047 | 0.464 | 1.00 | 2.661 | 2,257 |
| | NDE | BKMR-CMA | 100 | 1.133 | −0.044 | 0.027 | 0.272 | 0.96 | 1.114 | 92 |
| | | BART-CMA | 100 | 1.133 | 0.708 | 0.045 | 0.835 | 0.89 | 2.540 | 2,257 |
| | NIE | BKMR-CMA | 100 | 1.961 | −0.062 | 0.026 | 0.263 | 0.99 | 1.219 | 92 |
| | | BART-CMA | 100 | 1.961 | −0.746 | 0.027 | 0.792 | 0.52 | 1.418 | 2,257 |
| 18 | TE | BKMR-CMA | 100 | 3.092 | −0.065 | 0.023 | 0.236 | 0.91 | 0.897 | 90 |
| | | BART-CMA | 100 | 3.092 | −0.095 | 0.046 | 0.470 | 1.00 | 2.659 | 2,209 |
| | NDE | BKMR-CMA | 100 | 1.132 | −0.013 | 0.028 | 0.274 | 0.97 | 1.130 | 90 |
| | | BART-CMA | 100 | 1.132 | 0.638 | 0.046 | 0.787 | 0.89 | 2.532 | 2,209 |
| | NIE | BKMR-CMA | 100 | 1.960 | −0.052 | 0.024 | 0.245 | 0.98 | 1.248 | 90 |
| | | BART-CMA | 100 | 1.960 | −0.733 | 0.024 | 0.772 | 0.57 | 1.428 | 2,209 |
| 19 | TE | BKMR-CMA | 100 | 3.139 | −0.078 | 0.027 | 0.275 | 0.91 | 0.929 | 91 |
| | | BART-CMA | 100 | 3.139 | 0.071 | 0.063 | 0.632 | 0.99 | 2.929 | 1,350 |
| | NDE | BKMR-CMA | 100 | 1.181 | −0.099 | 0.026 | 0.275 | 0.98 | 1.165 | 91 |
| | | BART-CMA | 100 | 1.181 | 0.729 | 0.063 | 0.958 | 0.86 | 2.813 | 1,350 |
| | NIE | BKMR-CMA | 100 | 1.958 | 0.021 | 0.025 | 0.251 | 0.97 | 1.300 | 91 |
| | | BART-CMA | 100 | 1.958 | −0.658 | 0.029 | 0.719 | 0.64 | 1.562 | 1,350 |

**Table S2.** DeepMed performance under the quartile binarization of the mixture index. All estimates are scored against the truth defined for the componentwise 25th-to-75th-percentile mixture shift, so the reported bias reflects both estimation error and the mismatch between the binarized treatment contrast and the target estimand (Section 2.4 of the main text). Relative bias is the absolute bias as a percentage of the true value.

| Scenario | Estimand | n used | Reps | Truth | Estimate | Bias | Rel. bias (%) | Coverage | Width |
|---|---|---|---|---|---|---|---|---|---|
| 1 | TE | 150 | 100 | 2.548 | 1.939 | −0.610 | 23.9 | 0.10 | 0.734 |
| | NDE | 150 | 100 | 1.446 | 2.064 | 0.617 | 42.7 | 0.28 | 0.896 |
| | NIE | 150 | 100 | 1.102 | −0.586 | −1.687 | 153.2 | 0.00 | 0.410 |
| 2 | TE | 500 | 100 | 2.548 | 1.080 | −1.468 | 57.6 | 0.00 | 0.916 |
| | NDE | 500 | 100 | 1.446 | 0.948 | −0.498 | 34.5 | 0.64 | 1.287 |
| | NIE | 500 | 100 | 1.102 | −0.275 | −1.376 | 125.0 | 0.00 | 0.517 |
| 3 | TE | 150 | 100 | 2.897 | 1.926 | −0.971 | 33.5 | 0.02 | 0.962 |
| | NDE | 150 | 100 | 1.035 | 2.139 | 1.103 | 106.5 | 0.00 | 1.036 |
| | NIE | 150 | 100 | 1.862 | −0.815 | −2.677 | 143.8 | 0.00 | 0.543 |
| 4 | TE | 500 | 99 | 2.897 | 0.617 | −2.281 | 78.7 | 0.00 | 1.047 |
| | NDE | 500 | 99 | 1.035 | 0.639 | −0.397 | 38.3 | 0.79 | 1.293 |
| | NIE | 500 | 99 | 1.862 | −0.611 | −2.473 | 132.8 | 0.00 | 0.577 |
| 5 | TE | 150 | 100 | 2.690 | 1.801 | −0.890 | 33.1 | 0.01 | 0.800 |
| | NDE | 150 | 100 | 1.532 | 1.937 | 0.405 | 26.4 | 0.58 | 0.921 |
| | NIE | 150 | 100 | 1.158 | −0.603 | −1.761 | 152.1 | 0.00 | 0.447 |
| 6 | TE | 500 | 100 | 2.690 | 0.729 | −1.961 | 72.9 | 0.00 | 0.984 |
| | NDE | 500 | 100 | 1.532 | 0.695 | −0.837 | 54.6 | 0.29 | 1.337 |
| | NIE | 500 | 100 | 1.158 | −0.350 | −1.508 | 130.2 | 0.00 | 0.514 |
| 7 | TE | 150 | 99 | 3.088 | 1.884 | −1.204 | 39.0 | 0.01 | 1.091 |
| | NDE | 150 | 99 | 1.131 | 2.071 | 0.940 | 83.1 | 0.10 | 1.156 |
| | NIE | 150 | 99 | 1.958 | −0.856 | −2.814 | 143.7 | 0.00 | 0.604 |
| 8 | TE | 500 | 100 | 3.088 | 0.269 | −2.820 | 91.3 | 0.00 | 1.219 |
| | NDE | 500 | 100 | 1.131 | 0.312 | −0.819 | 72.4 | 0.37 | 1.516 |
| | NIE | 500 | 100 | 1.958 | −0.839 | −2.796 | 142.8 | 0.00 | 0.642 |

https://doi.org/10.3390/xxxxx

| Scenario | Estimand | n used | Reps | Truth | Estimate | Bias | Rel. bias (%) | Coverage | Width |
|---|---|---|---|---|---|---|---|---|---|
| 9 | TE | 150 | 100 | 0.395 | 0.229 | −0.166 | 42.0 | 0.17 | 0.205 |
| | NDE | 150 | 100 | 0.199 | 0.339 | 0.139 | 69.8 | 0.44 | 0.256 |
| | NIE | 150 | 100 | 0.195 | −0.070 | −0.265 | 135.6 | 0.00 | 0.133 |
| 10 | TE | 500 | 100 | 0.395 | 0.190 | −0.205 | 51.8 | 0.22 | 0.295 |
| | NDE | 500 | 100 | 0.199 | 0.176 | −0.024 | 11.8 | 0.94 | 0.457 |
| | NIE | 500 | 100 | 0.195 | 0.058 | −0.137 | 70.2 | 0.12 | 0.152 |
| 11 | TE | 150 | 100 | 2.640 | 1.780 | −0.860 | 32.6 | 0.07 | 1.034 |
| | NDE | 150 | 100 | 1.094 | 1.898 | 0.805 | 73.6 | 0.09 | 1.054 |
| | NIE | 150 | 100 | 1.546 | 0.661 | −0.885 | 57.2 | 0.00 | 0.366 |
| 12 | TE | 150 | 100 | 5.473 | 2.594 | −2.879 | 52.6 | 0.00 | 1.527 |
| | NDE | 150 | 100 | 0.840 | 2.591 | 1.751 | 208.6 | 0.00 | 1.409 |
| | NIE | 150 | 100 | 4.633 | 0.494 | −4.139 | 89.3 | 0.00 | 0.751 |

**Table S3.** Performance of BKMR-CMA with componentwise variable selection disabled in scenarios 1, 7, and 8 (sensitivity analysis for Section 3.1.6). Bias, RMSE and width are on the outcome scale; MCSE is the Monte Carlo standard error of the bias. Fits is the number of replications out of 100 that produced a usable estimate. Each scenario was analyzed on the same 100 datasets as in the primary analysis. Results with variable selection enabled, and for the binary-outcome scenarios 9–10 (in which selection was disabled), are given in Table S1.

| Scenario | Estimand | Method | Fits | Truth | Bias | MCSE | RMSE | Coverage | Width |
|---|---|---|---|---|---|---|---|---|---|
| 1 | TE | BKMR-CMA | 100 | 2.548 | 0.175 | 0.013 | 0.218 | 0.92 | 0.682 |
| | NDE | BKMR-CMA | 100 | 1.446 | 0.018 | 0.020 | 0.202 | 0.97 | 0.930 |
| | NIE | BKMR-CMA | 100 | 1.102 | 0.157 | 0.019 | 0.243 | 0.96 | 1.017 |
| 7 | TE | BKMR-CMA | 100 | 3.088 | 0.020 | 0.021 | 0.206 | 0.96 | 0.877 |
| | NDE | BKMR-CMA | 100 | 1.131 | 0.025 | 0.024 | 0.244 | 0.98 | 1.149 |
| | NIE | BKMR-CMA | 100 | 1.958 | −0.004 | 0.026 | 0.258 | 0.98 | 1.283 |
| 8 | TE | BKMR-CMA | 100 | 3.088 | −0.062 | 0.013 | 0.143 | 0.94 | 0.507 |
| | NDE | BKMR-CMA | 100 | 1.131 | −0.062 | 0.018 | 0.189 | 0.96 | 0.719 |
| | NIE | BKMR-CMA | 100 | 1.958 | −0.001 | 0.016 | 0.162 | 0.98 | 0.798 |

Runtimes are not reported because this arm was run separately from the primary analysis, under different cluster conditions, and is not comparable with the runtimes in Table S1.

**Table S4.** Decomposition of the DeepMed bias in the natural indirect effect into estimand mismatch and estimation error. Each binarization is scored against two truths: the target truth for the componentwise $Q_{25} \rightarrow Q_{75}$ shift, and the truth for the contrast the binarization implies (own contrast).

| Scenario | Split | Mean contrast | Truth (target) | Truth (own) | Mismatch | Estimate | Bias vs. target (MCSE) | Estimation error | Coverage (target) | Coverage (own) |
|---|---|---|---|---|---|---|---|---|---|---|
| 1 | Median | 1.108 | 1.102 | 0.886 | −0.215 | −0.243 | −1.345 (0.010) | −1.129 | 0.00 | 0.00 |
| | Quartile | 1.765 | 1.102 | 1.412 | 0.310 | −0.586 | −1.687 (0.020) | −1.997 | 0.00 | 0.00 |
| 2 | Median | 1.108 | 1.102 | 0.886 | −0.215 | −0.049 | −1.151 (0.014) | −0.935 | 0.00 | 0.00 |
| | Quartile | 1.765 | 1.102 | 1.412 | 0.310 | −0.275 | −1.376 (0.015) | −1.687 | 0.00 | 0.00 |
| 3 | Median | 1.108 | 1.862 | 1.379 | −0.483 | −0.326 | −2.188 (0.011) | −1.705 | 0.00 | 0.00 |
| | Quartile | 1.765 | 1.862 | 2.661 | 0.799 | −0.815 | −2.677 (0.022) | −3.475 | 0.00 | 0.00 |
| 4 | Median | 1.108 | 1.862 | 1.379 | −0.483 | −0.124 | −1.985 (0.014) | −1.503 | 0.00 | 0.00 |
| | Quartile | 1.765 | 1.862 | 2.661 | 0.799 | −0.611 | −2.473 (0.019) | −3.272 | 0.00 | 0.00 |
| 5 | Median | 1.108 | 1.158 | 0.963 | −0.195 | −0.260 | −1.418 (0.011) | −1.223 | 0.00 | 0.00 |
| | Quartile | 1.765 | 1.158 | 1.435 | 0.277 | −0.603 | −1.761 (0.020) | −2.038 | 0.00 | 0.00 |
| 6 | Median | 1.108 | 1.158 | 0.963 | −0.195 | −0.107 | −1.265 (0.015) | −1.070 | 0.00 | 0.00 |
| | Quartile | 1.765 | 1.158 | 1.435 | 0.277 | −0.350 | −1.508 (0.017) | −1.785 | 0.00 | 0.00 |
| 7 | Median | 1.108 | 1.958 | 1.498 | −0.460 | −0.405 | −2.363 (0.013) | −1.903 | 0.00 | 0.00 |
| | Quartile | 1.765 | 1.958 | 2.704 | 0.747 | −0.856 | −2.814 (0.024) | −3.561 | 0.00 | 0.00 |
| 8 | Median | 1.108 | 1.958 | 1.498 | −0.460 | −0.230 | −2.187 (0.014) | −1.728 | 0.00 | 0.00 |
| | Quartile | 1.765 | 1.958 | 2.704 | 0.747 | −0.839 | −2.796 (0.020) | −3.543 | 0.00 | 0.00 |
| 9 | Median | 1.108 | 0.195 | 0.168 | −0.027 | 0.010 | −0.185 (0.003) | −0.158 | 0.00 | 0.00 |
| | Quartile | 1.765 | 0.195 | 0.227 | 0.031 | −0.070 | −0.265 (0.007) | −0.296 | 0.00 | 0.00 |
| 10 | Median | 1.108 | 0.195 | 0.168 | −0.027 | 0.040 | −0.155 (0.004) | −0.128 | 0.07 | 0.18 |
| | Quartile | 1.765 | 0.195 | 0.227 | 0.031 | 0.058 | −0.137 (0.005) | −0.169 | 0.12 | 0.06 |
| 11 | Median | 1.211 | 1.546 | 1.326 | −0.220 | 0.222 | −1.324 (0.010) | −1.104 | 0.00 | 0.00 |
| | Quartile | 1.929 | 1.546 | 2.359 | 0.813 | 0.661 | −0.885 (0.018) | −1.698 | 0.00 | 0.00 |
| 12 | Median | 1.025 | 4.633 | 3.034 | −1.599 | 0.105 | −4.529 (0.021) | −2.930 | 0.00 | 0.00 |
| | Quartile | 1.633 | 4.633 | 5.974 | 1.341 | 0.494 | −4.139 (0.028) | −5.480 | 0.00 | 0.00 |

Split: median split or quartile binarization of the mixture index (Section 2.4). Mean contrast: average componentwise difference between the upper-group and lower-group mean exposure vectors; the target $Q_{25} \rightarrow Q_{75}$ contrast is 1.38 for every exposure. Truths are computed from the same $10^6$ simulated observations. Mismatch = truth (own contrast) − truth (target); estimation error = estimate − truth (own contrast); bias vs. target = mismatch + estimation error. MCSE, Monte Carlo standard error of the estimate. Coverage refers to the nominal 95% confidence interval. Scenarios 9–10 have binary outcomes, so their effects are risk differences. Each cell is based on 99–100 replicates.

**Table S5. Cross-world support diagnostics for the seventeen synthetic scenarios, the two plasmode cells and the NHANES analytic sample (Section 2.5.1 of the main text; Figure 2).**

| Setting | n | Mixture–mediator correlation | Scalar cross-world occupancy (%) | Cross-world points outside support (%) | Factual points outside support (%) |
|---|---|---|---|---|---|
| Scenario 1 | 300 | 0.72 | 0.45 | 11.30 [2.49, 24.89] | 0.13 / 0.11 |
| Scenario 2 | 1000 | 0.72 | 0.44 | 10.08 [4.04, 18.01] | 0.08 / 0.08 |
| Scenario 3 | 300 | 0.72 | 0.46 | 10.56 [3.00, 23.51] | 0.11 / 0.07 |
| Scenario 4 | 1000 | 0.72 | 0.45 | 9.41 [3.35, 16.61] | 0.09 / 0.06 |
| Scenario 5 | 300 | 0.70 | 0.53 | 10.84 [3.33, 20.67] | 0.08 / 0.06 |
| Scenario 6 | 1000 | 0.70 | 0.53 | 10.05 [4.28, 17.14] | 0.07 / 0.07 |
| Scenario 7 | 300 | 0.70 | 0.50 | 10.69 [2.98, 21.08] | 0.06 / 0.10 |
| Scenario 8 | 1000 | 0.70 | 0.56 | 10.25 [4.39, 18.57] | 0.07 / 0.07 |
| Scenario 9 | 300 | 0.69 | 0.54 | 10.37 [2.49, 21.70] | 0.05 / 0.08 |
| Scenario 10 | 1000 | 0.70 | 0.56 | 9.64 [4.10, 16.82] | 0.07 / 0.06 |
| Scenario 11 | 300 | 0.66 | 0.78 | 11.25 [2.67, 22.18] | 0.33 / 0.30 |
| Scenario 12 | 300 | 0.85 | 0.04 | 32.03 [12.63, 52.05] | 0.00 / 0.01 |
| Scenario 15 | 300 | 0.61 | 0.98 | 5.20 [0.82, 13.56] | 0.08 / 0.08 |
| Scenario 16 | 300 | 0.57 | 1.42 | 0.56 [0.00, 2.00] | 0.00 / 0.00 |
| Scenario 17 | 300 | 0.79 | 0.14 | 9.02 [2.00, 16.18] | 0.06 / 0.06 |
| Scenario 18 | 300 | 0.68 | 0.68 | 9.57 [2.49, 19.68] | 0.07 / 0.07 |
| Scenario 19 | 300 | 0.68 | 0.61 | 8.85 [2.32, 19.84] | 0.03 / 0.06 |
| Scenario 13 (plasmode) | 1000 | −0.05 | 6.76 | 0.06 [0.00, 0.30] | 0.06 / 0.04 |
| Scenario 14 (plasmode) | 1000 | −0.06 | 7.20 | 0.05 [0.00, 0.20] | 0.05 / 0.04 |
| NHANES (20 imputations) | 1498 | −0.06 | 6.79 | 0.22 [0.03, 0.40] | 0.12 / 0.14 |

Mixture–mediator correlation: Pearson correlation between the mixture index, the sum of the standardized exposures, and the mediator. Scalar cross-world occupancy: percentage of observations lying jointly in the upper quartile of the mixture index and the lower quartile of the mediator. Cross-world points outside support: percentage of points ($z_1$, $m_0$) whose distance to its fifth-nearest observed point, in the whitened exposure–mediator space, exceeded the 95th percentile of the same leave-one-out distance among the observed points; about 5% of points drawn from the observed distribution are classified as outside by construction. Factual points outside support: the same quantity computed at ($z_1$, $m_1$) and at ($z_0$, $m_0$), reported in that order. Brackets give the 2.5th and 97.5th percentiles across replications. Synthetic cells are based on 100 replications, plasmode cells on 100 replications and the applied column on the 20 imputed datasets.

## Supplementary Figures

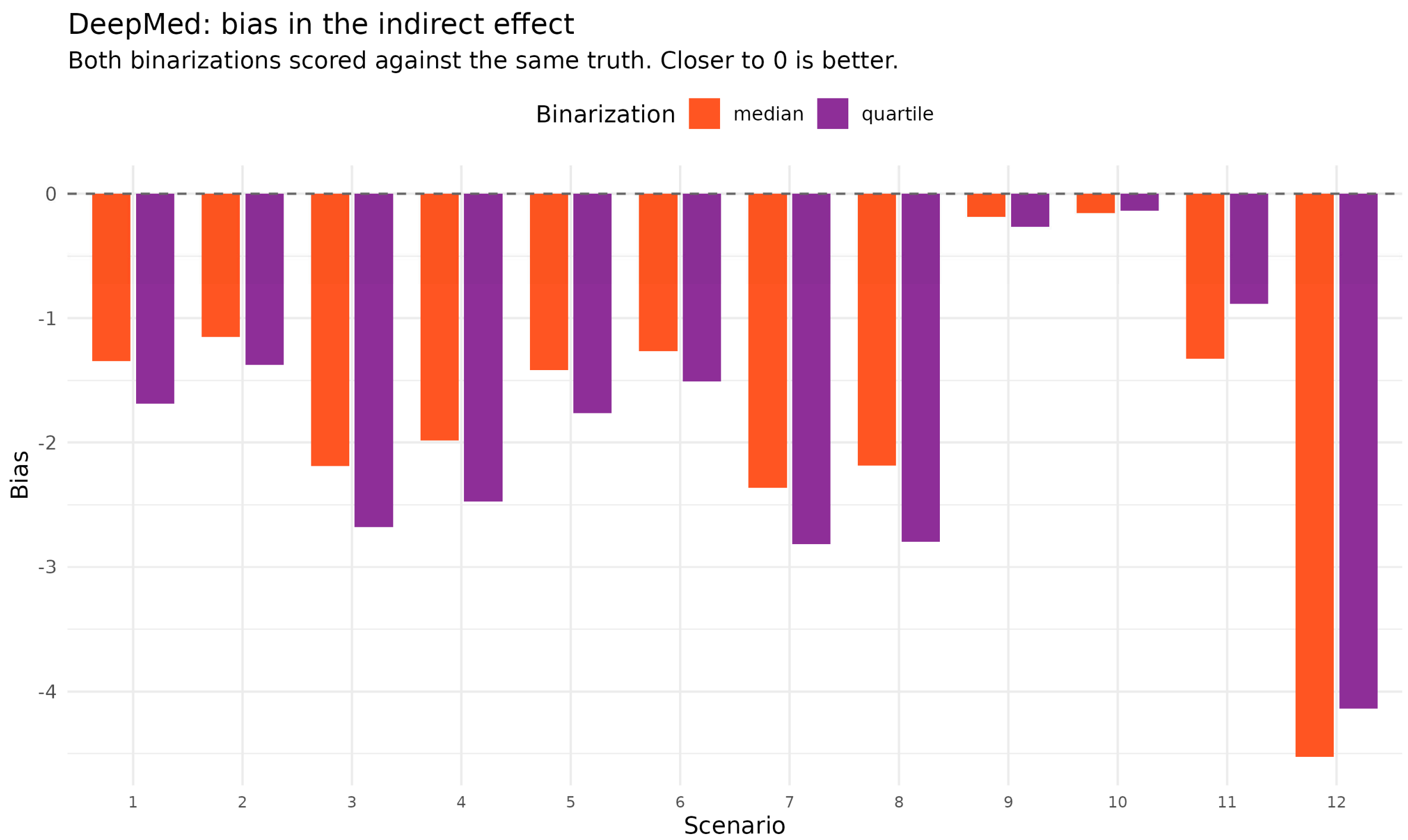


**Figure S1.** DeepMed: bias in the natural indirect effect across the twelve synthetic scenarios. Both binarizations of the mixture index are scored against the same truth, defined for the componentwise 25th-to-75th-percentile mixture shift. Values closer to zero indicate better performance.

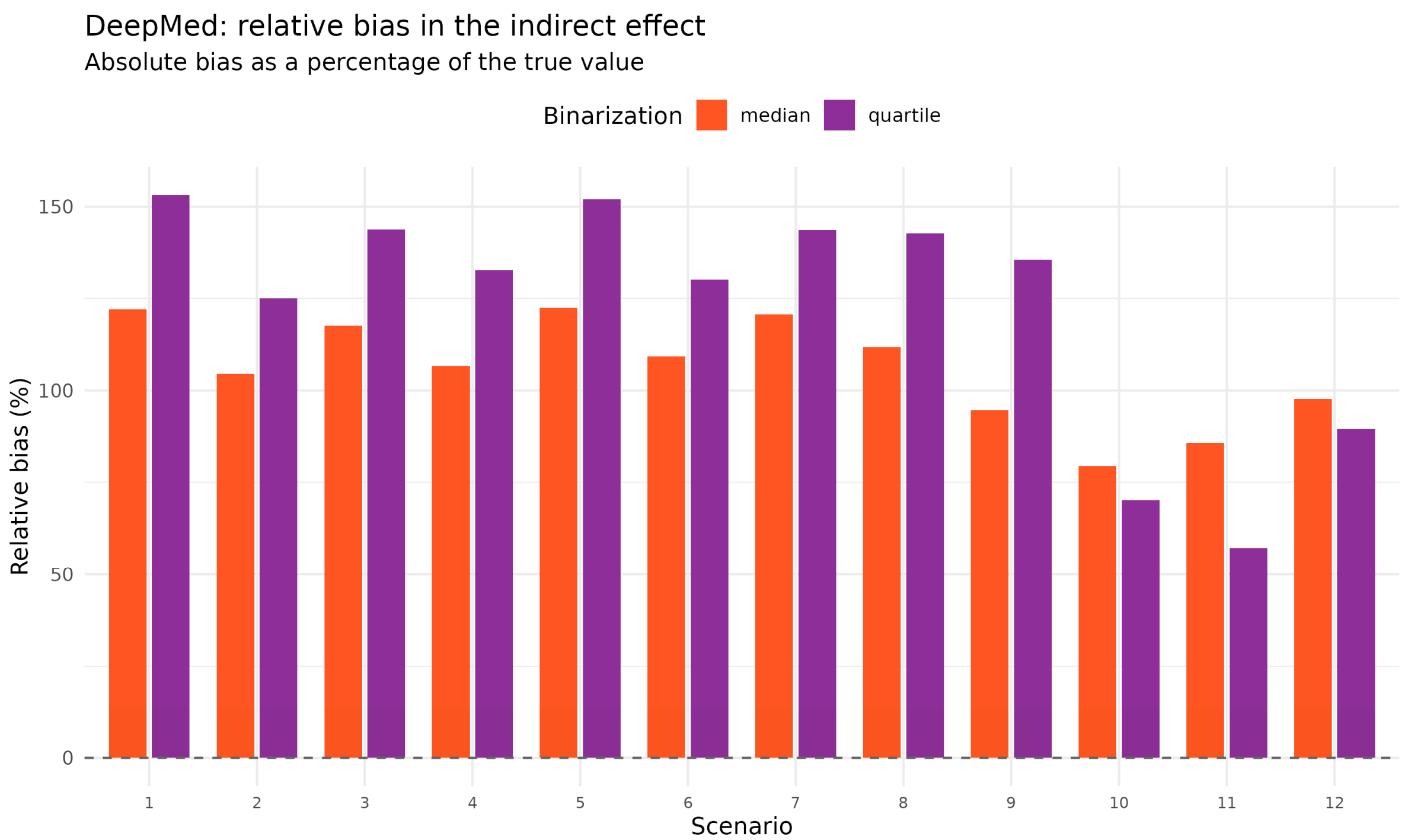


**Figure S2.** DeepMed: relative bias in the natural indirect effect, expressed as the absolute bias as a percentage of the true value.

https://doi.org/10.3390/xxxxx

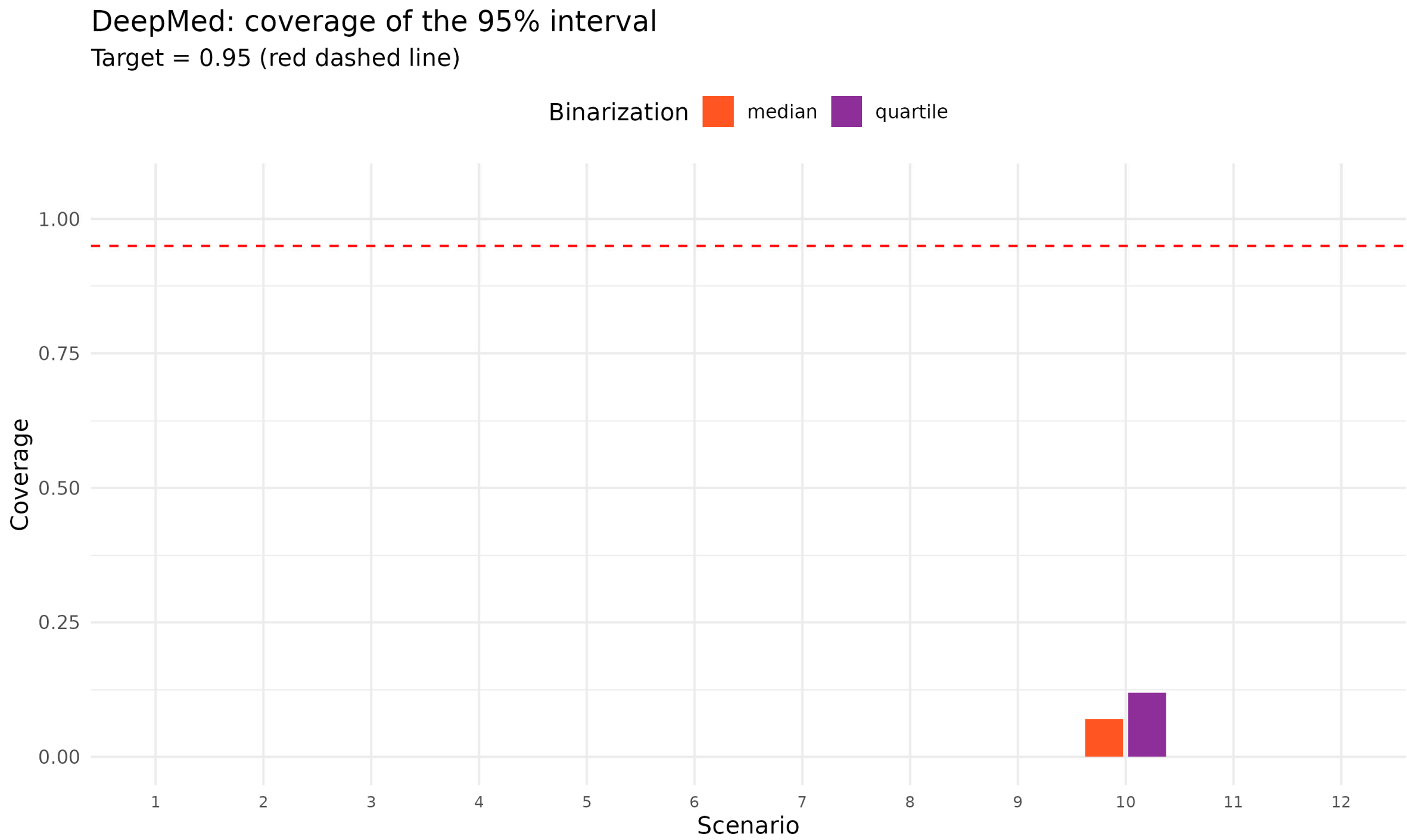


**Figure S3.** DeepMed: coverage of the nominal 95% confidence interval for the natural indirect effect. The dashed line marks the 0.95 target. Coverage is zero in eleven of the twelve scenarios under both binarizations.

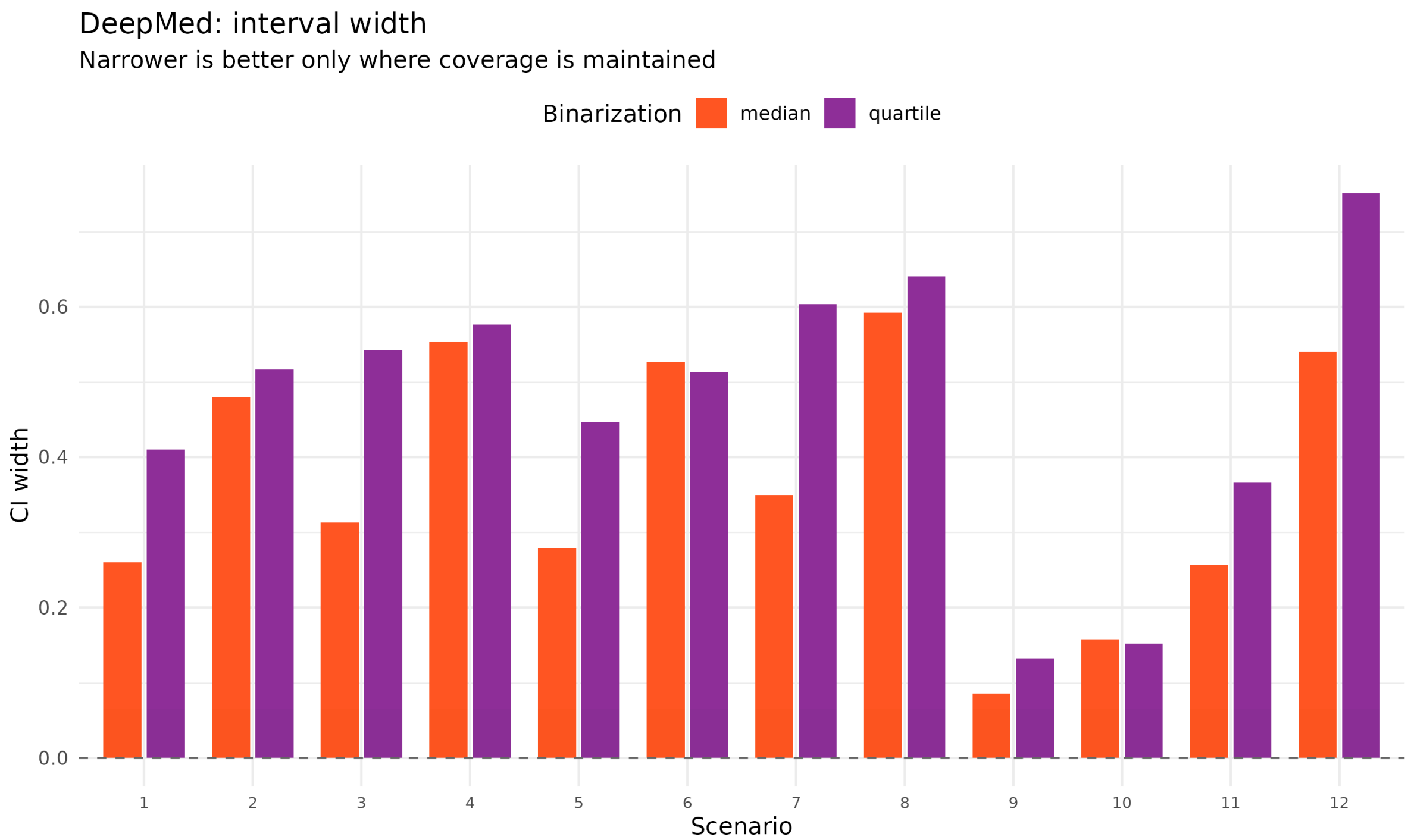


**Figure S4.** DeepMed: mean width of the 95% confidence interval for the natural indirect effect. Narrower intervals indicate greater precision only where coverage is maintained (Figure S3).

https://doi.org/10.3390/xxxxx

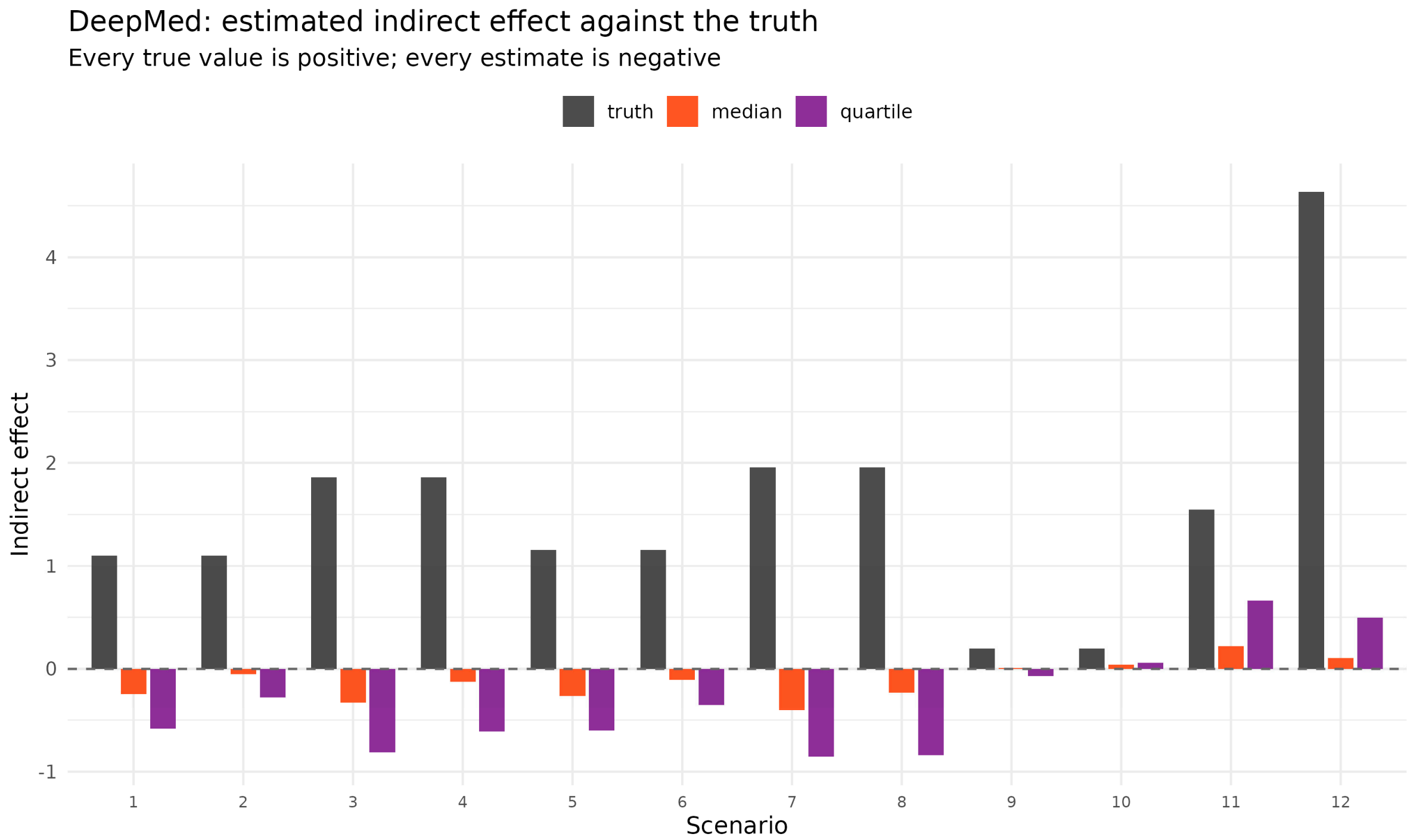


**Figure S5. DeepMed: estimated indirect effect against the truth. The true natural indirect effect is positive in every scenario. Every estimate is negative in the eight core scenarios under both binarizations; in scenarios 9–12 the estimates are mostly positive but far below the truth.**

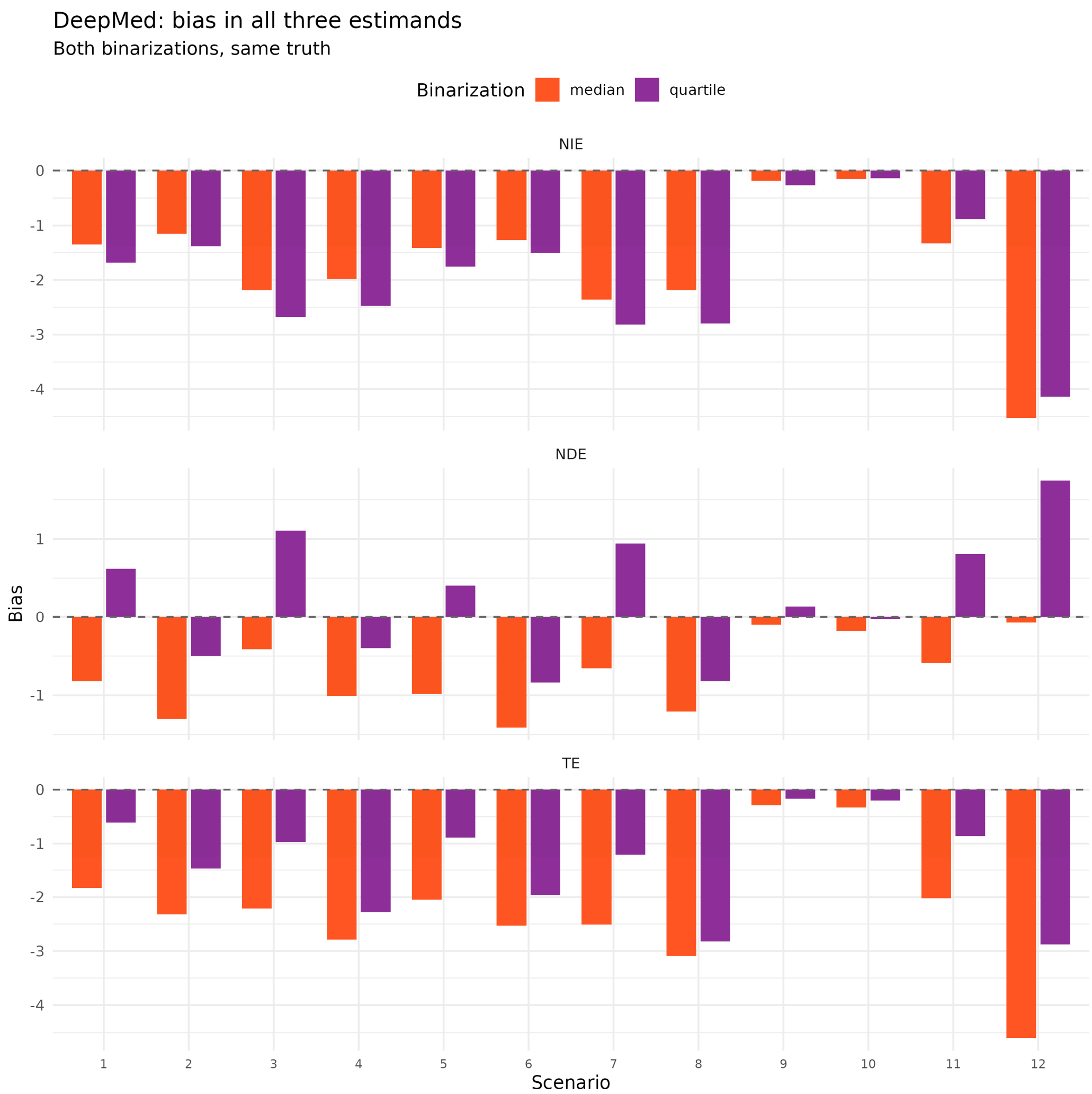


https://doi.org/10.3390/xxxxx

**Figure S6.** DeepMed: bias in all three estimands (natural indirect effect, natural direct effect and total effect) under both binarizations.

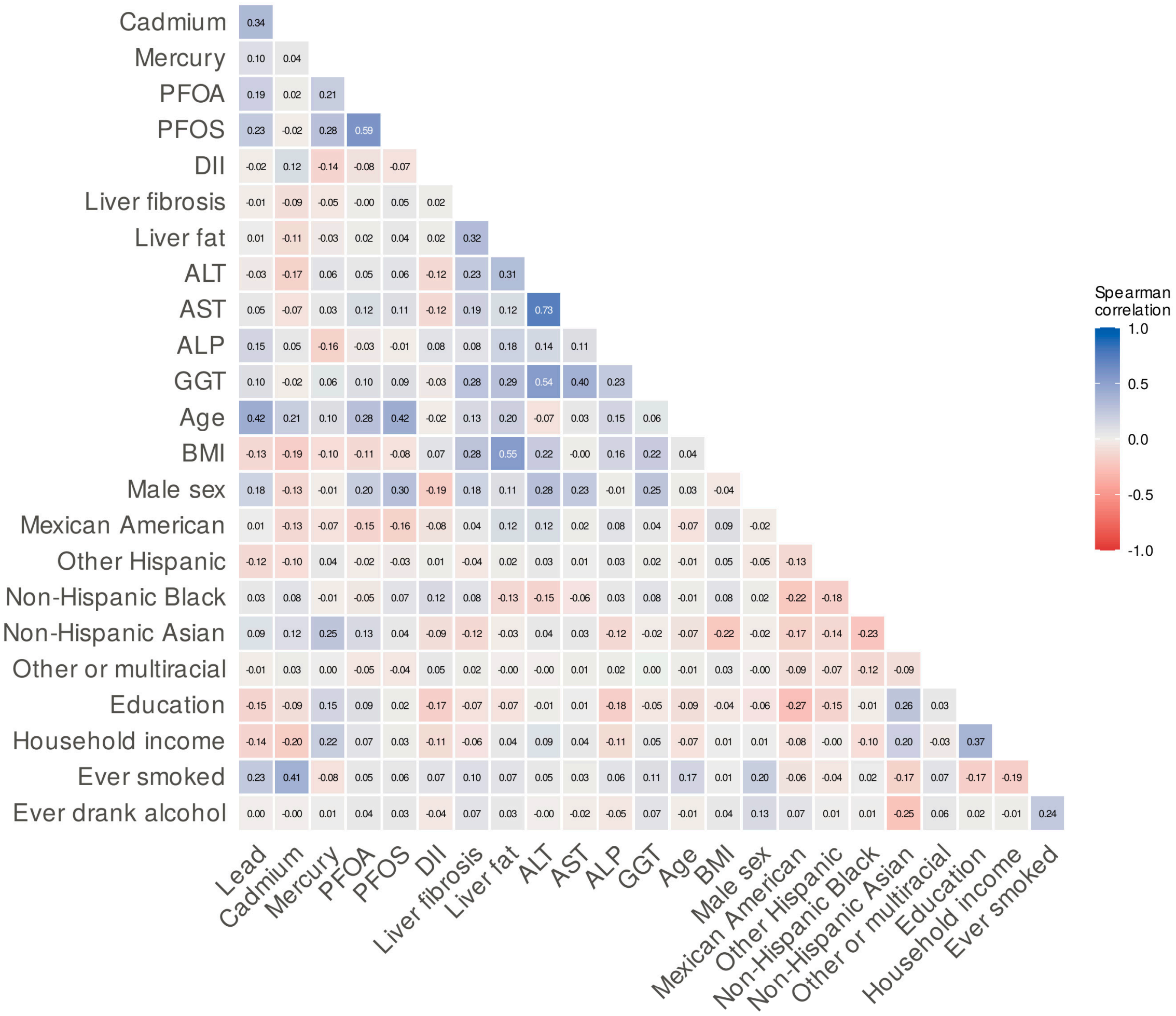


**Figure S7. Spearman correlation matrix for the primary confounder set: the log-transformed exposure biomarkers, the Dietary Inflammatory Index, the six liver outcomes and the demographic and behavioural covariates used in the primary adjustment (age, BMI, sex, race and ethnicity, education, household income, smoking and alcohol use). The downstream cardiometabolic covariates adjusted for in the sensitivity analysis are not shown. Because Spearman correlation is rank-based, the monotonic log transformation applied to the exposure biomarkers does not alter these coefficients. Correlations are pooled across the 20 imputed datasets (n = 1498).**
